# A Review of Patient-Reported Outcome Measures in Childhood Cancer

**DOI:** 10.3390/children9101497

**Published:** 2022-09-30

**Authors:** Madeline R. Horan, Jin-ah Sim, Kevin R. Krull, Justin N. Baker, I-Chan Huang

**Affiliations:** 1 Department of Epidemiology and Cancer Control, St. Jude Children’s Research Hospital, Memphis, TN 38105, USA; 2 School of AI Convergence, Hallym University, Chuncheon 200160, Korea; 3 Department of Psychology, St. Jude Children’s Research Hospital, Memphis, TN 38105, USA; 4 Department of Oncology, St. Jude Children’s Research Hospital, Memphis, TN 38105, USA

**Keywords:** childhood cancer survivors, chronic health conditions, health-related quality-of-life, symptoms

## Abstract

Patient-reported outcomes (PROs) are used in clinical work and research to capture the subjective experiences of childhood cancer patients and survivors. PROs encompass content domains relevant and important to this population, including health-related quality-of-life (HRQOL), symptoms, and functional status. To inform future efforts in the application of PRO measures, this review describes the existing generic and cancer-specific PRO measures for pediatric cancer populations and summarizes their characteristics, available language translations, content coverage, and measurement properties into tables for clinicians and researchers to reference before choosing a PRO measure that suits their purpose. We have identified often unreported measurement properties that could provide evidence about the clinical utility of the PRO measures. Routine PRO assessment in pediatric cancer care offers opportunities to facilitate clinical decision-making and improve quality of care for these patients. However, we suggest that before implementing PRO measures into research or clinical care, the psychometric properties and content coverage of the PRO measures must be considered to ensure that PRO measures are appropriately assessing the intended construct in childhood cancer patients.

## 1. Introduction

With advances in cancer treatments, the survival rate of children and adolescents with cancer is now over 80% in many high-income countries and 37% globally [1]. However, childhood cancer patients and survivors may experience many different acute and chronic physical and psychological side effects (e.g., anemia, constipation, fatigue, pain) and late effects (e.g., cognitive impairment, neuropathy, hearing loss, cardiac, pulmonary, and endocrine disorders) from their cancer diagnosis and associated treatments [2]. Over the course of their cancer journey from diagnosis to survivorship, these effects are likely to impact the patient’s/survivor’s health-related quality-of-life (HRQOL), differentially for patients with different diagnoses and treatments [3]. Overall, patients and survivors report poorer HRQOL and daily functioning [4,5] and increased symptom burden [6] compared to their siblings and the general population. The subjective nature of many of these acute side effects and late effects are well-suited to be assessed via patient-reported outcome (PRO) measures. PROs is an umbrella term to capture the concepts of HRQOL, functional status, subjective well-being, and physical, somatic, and psychological symptoms [7].

The subjective experience of childhood cancer patients and survivors is an important outcome to consider in survivorship research, patient and survivorship care, and clinical trials. As of 2009, the US FDA requires that all clinical trials use PRO measures as an efficacy endpoint [8], but only 21% of clinical trials conducted from 2007 to 2020 that included adolescents and young adults with cancer collected PRO measures as a secondary or exploratory endpoint [9]. PRO measures have been shown to better predict survival than clinician-assessed performance and biomarkers in adult cancer trials [10]. In pediatric cancer survivors, PRO measures of symptom burden have predicted the progression of late effect CHCs [11]. and comorbid symptoms have been shown to affect HRQOL [12,13,14] and adverse health outcomes [15,16,17]. Even though PRO measures play an important role in pediatric oncology, clinicians report barriers to using these measures in their practices, such as the lack of training to administer, score, and interpret PROs, the lack of clinically meaningful cutpoints to aid in interpretation, and uncertainty about the validity of various PRO measures [18,19].

There have been published systematic reviews of HRQOL measures for childhood cancer patient and survivor populations [3,20,21,22,23,24,25], but these review studies tend to summarize the measurement properties rather than evaluate the content coverage of PRO measures and identify specific measurement properties of PRO measures that require further improvement for clinical use. This review describes the extant PRO measures for pediatric cancer populations and summarizes their characteristics, content coverage, and measurement properties. The PRO measures that we selected are for patients/survivors younger than 21 years of age, but the clinical application (e.g., predictive validity for late effects) can be extended to adult survivors of childhood cancer. In this review, we aimed to identify measurement properties of PRO measures that are relevant to clinical practice, discuss the ways in which these measures and their clinical application can be improved, and provide recommendations for future implementation of PRO measures.

## 2. Materials and Methods

**Figure 1:**
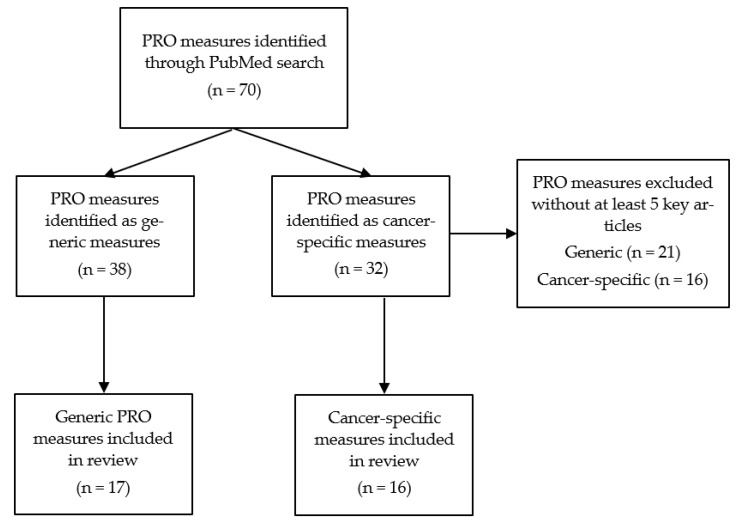
Flow diagram of PRO measure selection.

In 2019, we searched PubMed for existing review articles of PRO measures to identify existing measures (see Figure 1 for a flow diagram of PRO measure selection). Then, we searched PubMed for additional PRO measures. For each PRO measure, we searched PubMed with the name and abbreviations of the measure (e.g., “Pediatric Quality of Life Inventory” OR “PedsQL”) and the following search terms: (cancer [title/abstract] OR tumor [title/abstract] OR tumors [title/abstract] OR oncolog* [title/abstract] OR chemoth* [title/abstract] OR “bone marrow transplantation” [title/abstract] OR “stem cell transplantation” [title/abstract]) AND (pediatric [title/abstract] OR paediatric [title/abstract] OR child [title/abstract] OR childhood [title/abstract] OR children [title/abstract] OR youth [title/abstract] OR adolescent* [title/abstract] OR “young adult” [title/abstract]). Articles were considered for inclusion if the full text was available, the article and PRO measure were available in English, the population was childhood cancer patients and/or survivors, and the study sample was less than 21 years old. Conference abstracts and grey literature were excluded. The article abstracts were reviewed for psychometric and clinical application information. Key articles about the psychometric properties were selected if the article was from a peer-reviewed journal, reported at least one of the psychometric properties from the COSMIN user manual [26,27,28], and the measure was externally validated with a sample different from the one used in the original development of the PRO measure. PRO measures with at least five key published articles were retained in this review to provide a summary of the most used and validated PRO measures. The PRO measures were then categorized as a generic or cancer-specific measure. The full and abbreviated names of each included PRO measure are provided in Table 1.

We then identified the original development articles, the article with the first or most representative application in childhood cancer patients/survivors, and the PRO measure’s website (if available) for each PRO measure. The characteristics of the PRO measure and the sample of childhood cancer patients/survivors from the first or most representative application of the PRO in this population were extracted (Supplementary Table S1). In addition, two different reviewers extracted and categorized the content coverage of each PRO measure based on existing classifications of content domains [20,21,29,30] (Table 2 and Table 3). Psychometric properties based on the COSMIN guidelines [26,27] were extracted from key validation articles (Table 4 and Supplementary Table S2).

## 3. Results

### 3.1. Generic PRO Measures

Seventeen generic PRO measures were identified through our literature search (see Supplementary Table S1 for study characteristics). The majority of the generic PRO measures use a Likert-type response scale. The HUI, PPS, and RCMAS all use binary response scales (e.g., yes/no). The CHIP and FPSR use a visual response scale (e.g., cartoon-depicted faces of different emotions for the patient to choose from). The number of items in each PRO measure varies greatly—from 1 visual response scale item in the PPS and FPSR to 602 items in all of the item banks of the PROMIS. PROs can be assessed through two primary approaches: health profile and health preference methods. Health profile measures provide scores for a patient’s perception on a variety of different PRO domains (e.g., physical functioning, psychological functioning) [31]. In contrast, health preference measures provide a single summary score of the patient’s perception of a PRO construct that is weighted by the general public’s valuation of different health states [31]. All but one generic PRO measure, the HUI, were created using the health profile approach. The HUI was developed to assess health status using the health preference approach. Many of the self-reported PRO measures are appropriate for children 8–17.9 years of age; some measures are appropriate for adolescents/young adults (e.g., BDI, HADS); and few measures (e.g., PPS, PedsQL-Core, KINDL) are available for younger children (e.g., 2–4.9 years of age) via parent-proxy reporting. Most measures have a recall period of at least one week, but some (e.g., state version of the STAIC, FPSR) are used for assessing the respondent’s current PRO status. Most of the generic PRO measures are available in various languages (see Supplementary Table S3 for available language translations).

These generic PRO measures have been widely used in studies of childhood cancer patients/survivors. For each measure, we identified the first or most representative study to administer the measure to either childhood cancer patients or survivors and summarized the sample characteristics in Supplementary Table S1. Twelve studies included cancer patients as the study population, and five studies assessed survivors. Most of the studies assessed adolescents, although a few studies measured PROs in children as young as 11 months (e.g., HUI). Many of the studies were conducted in the US, but there are also studies from Australia, Sweden, Canada, Austria, and the Netherlands. Most studies assessed children with a variety of cancer diagnoses, but a few included only children with leukemia, only children with CNS tumors, or only children with solid tumors.

### 3.2. Cancer-Specific PRO Measures

Sixteen cancer-specific PRO measures were identified through our search. Most employ a Likert-type response scale, but the ChIMES, OMDQ, Pain Squad App, and PeNAT all used visual scales. The number of items range from 6 to 48 across the cancer-specific measures. All of the cancer-specific PRO measures are health profile measures. Most measures are available for children 8–17.9 years of age, some for children 5–7.9 years old, and few for children under 5 years old. Most measures are available in both self-reported and proxy-reported forms. Many of the measures assess current PRO status of the patient/survivor, but some measures have a recall period of 24 h (e.g., OMDQ), one week (e.g., MSAS, PedsQL acute modules), two weeks (e.g., POQOLS), or one month (e.g., PCQL-32, PedsQL chronic modules).

The majority of the cancer-specific PRO measures were developed exclusively for childhood cancer patients/survivors, with the exception of the MSAS and OMDQ, which were first developed for adult cancer patients/survivors and then validated in pediatric cancer populations. In the studies that applied these cancer-specific measures to pediatric cancer patients/survivors, four assessed PROs in survivors via the PCQL-32, PEDQOL, PedsFACT-Brs, and PedsQL-Cancer, and the rest measured PROs in cancer patients currently undergoing treatment or newly off treatment. Most studies included both children and adolescents. All but four studies were conducted in the US. Three studies were conducted in Canada and one in Germany. All the studies assessed patients and/or survivors with a variety of cancer diagnoses except the brain tumor-specific measures (i.e., PedsFACT-Brs and PedsQL-Brain Tumor).

### 3.3. Measurement Properties

To better understand the limitations of the extant PRO measures, we examined their content coverage, scoring methods, and psychometric properties (e.g., cutpoints and minimally important differences [MIDs], responsiveness and predictive validity, and response-shift effects).

#### 3.3.1. Content Coverage

The content coverage of the generic PRO measures is listed in Table 2. The framework used for organizing the content domains is based on previously published reviews [20,30]. The generic PRO measures cover a wide variety of content domains. The most common domains are physical functioning, physical symptoms, and emotional distress. Body image is assessed by only two measures (i.e., BDI and PedsQL-Core). The measures that cover the greatest range of content areas across physical, psychological, and social domains are the BDI (9 areas), CHIP (11 areas), and CHQ (9 areas).

Many of the cancer-specific measures also cover physical and social health domains, but do not commonly assess domains of psychological health (see Table 3). Behavior is only assessed through the BASES. Of the cancer-specific measures, the most content domains are covered by the BASES (6 areas), MMQL (7 areas), and PEDQOL (6 areas). Five of the measures, the PCQL-32, PedsFACT-Brs, PedsQL-Brain Tumor, PedsQL Cancer, and POQOLS, cover PRO content related to the cancer diagnosis and treatment procedures (e.g., brain tumor survivor-specific concerns, disease and treatment-related symptoms, disease specific modules, procedural anxiety, reaction to current medical treatment, treatment anxiety).

#### 3.3.2. Scoring Methods

Most of the extant PRO measures employ a summation method to calculate PRO domain scores (e.g., PedsQL-Core, BASES). Recent PRO measures (e.g., PROMIS) use more rigorous methods (e.g., Item Response Theory or Confirmatory Factor Analysis weights) by taking into account possible differences in the relationship between the construct of interest and individual items [32,33,34].

#### 3.3.3. Psychometric Properties

Psychometric criteria were not fully tested in extant PRO measures. The majority of the generic and cancer-specific PRO measures have reported internal consistency, reliability, and construct or known-group validity. Many of the generic PRO measures also reported structural validity and cross-cultural validity or measurement invariance. According to the COSMIN guidelines, criterion validity requires a gold standard measure to be associated with the PRO measure, and the guidelines also indicate that there are no gold standard PRO measures [26]. Alternatively, a correlation between a short-form version and long-form version of the same PRO measure can be considered evidence for criterion validity [26], and this was reported for only two of the generic PRO measures (i.e., the BDI and KINDL) and none of the cancer-specific measures. Fewer psychometric properties related to clinical parameters (i.e., clinical validity) were assessed and/or reported for the cancer-specific measures compared to the generic PRO measures. These clinically relevant measurement properties that may facilitate clinical decision-making include cutpoints, MIDs, responsiveness to change, and response-shift effects.

Only four of the PRO measures (i.e., BDI, KIDSCREEN, PedsQL-Core, PROMIS) have established severity cutpoints and/or MIDs, and these properties are not available for any cancer-specific measures. Responsiveness to change, predictive validity, and response-shift effects are all uncommonly described in the literature addressing psychometric properties of existing PRO measures. All three of these properties are clinically useful for communication about the disease progression with patients and caregivers, facilitating decision-making about changing the treatment regimen, and tracking health status changes (e.g., disease progression, changes due to interventions) over time. Responsiveness to change was examined for seven of the generic PRO measures and seven of the cancer-specific PRO measures. Only one study examined response-shift effects using the PedsQL-Core [35].

## 4. Discussion

Concurrently capturing the content domains that are important to childhood cancer patients and examining the PRO measures’ psychometric properties is vitally important when implementing PRO measures into clinical care [29]. Our review examined both of these attributes of existing PRO measures in childhood cancer survivors. The extant PRO measures for this population are varied in their content coverage and reported measurement properties. Physical functioning, physical symptoms, and emotional distress are common content areas in the existing PRO measures. School functioning and self-esteem are content domains that are critically important to pediatric populations especially among adolescents, and they are not covered by any cancer-specific PRO measure but are available with some generic PRO measures. None of the PRO measures are comprehensive in their content coverage. Additionally, none of the existing PRO measures identified through our search assess other content domains relevant to childhood cancer patients and survivors, including cancer-related stigma, infertility concerns, chronic symptoms, future health expectations, and caregiver quality-of-life [29].

Some previous review studies have identified the measurement properties of extant PRO measures [22,25], and other reviews have identified uncertainty about the clinical validity of PRO measures as an implementation barrier [18,19]. To bridge these review topics, we have identified often unreported measurement properties that could provide evidence about the clinical utility of the PRO measures including clinical anchors, clinically meaningful cutpoints and MIDs, responsiveness, predictive validity, response-shift effects, and cross-cultural validity. Assessing clinical validity based on meaningful clinical anchors or known-groups in the development and validation of PRO measures is often under-reported. If known-group validity was assessed, most of the studies used extant measures to compare cancer patients/survivors (or cancer diagnoses) to healthy controls or siblings. However, most studies did not consider treatment modality data, objectively-evaluated physical or neurocognitive performance, or severity graded late effects or CHCs as clinical anchors for assessing clinical validation, which are known to affect PROs in childhood cancer patients and survivors [36,37]. Using and reporting on these clinical anchors may enhance the clinical utility of PRO measures.

In addition, clinicians report the lack of clinically meaningful cutpoints and MIDs as another application barrier [18,19]. Only four of the extant generic PRO measures included in our review established cutpoints and/or MIDs. Clinicians may want to use cutpoints to aid in the interpretation of the PRO score. For example, cutpoints are useful in a screening measure to identify patients or survivors at high levels of reported adverse health outcomes. MIDs are useful for clinicians to identify meaningful intraindividual change in their patients. Clinical anchors (e.g., change in number of fatigue episodes, change in performance on a cognitive task) can be used to establish MIDs that may facilitate clinical interpretability of PRO scores [8]. MIDs should be calculated based on patient-centered and/or clinically based anchors so that differences in scores that reach this MID threshold have patient-centered meaning. MIDs are typically estimated with clinical anchors or distribution-based methods, but there are some novel approaches to estimating MIDs, such as scale judgment [38] and bookmarking [39], that require patients, family members, and health care professionals to judge different health scenario vignettes. Then IRT methods are used to identify the MIDs from their judgments. Similar to traditional clinical anchors, these are patient-centered approaches to establishing MIDs, but they also incorporate judgements from multiple perspectives.

Other under-reported measurement properties related to clinical validity are responsiveness, predictive validity, and response-shift effects. Clinical anchors can be used to assess responsiveness, which is the ability of a PRO measure to detect underlying health status changes over time. A related property is predictive validity, which uses baseline or the change pattern of PROs to predict adverse health events or disease progression, including premature mortality. Response-shift effects capture changes in a patient/survivor’s responses to a PRO measure that reflect shifts in the person’s values, standards, or conceptualization of the PRO and their psychological adaptation during their cancer journey. Without knowing the magnitude of response shift, the comparisons of PRO scores before and after the interventions or disease activities will be biased as will other longitudinal assessments. These time-dependent properties are clinically useful for predicting disease progression, advising preventive interventions, and longitudinal research of PROs.

Many of the extant PRO measures are available in multiple languages. However, few have been extensively cross-culturally validated. Childhood cancer is a rare disease, so clinical trials often need to pool different participants across nations [40]. This requires multiple language translations of the same PRO measure, and cross-cultural validity provides evidence that the items are assessing the same construct in the same way across language translations [26]. The COSMIN guidelines require two groups (e.g., a Chinese-speaking sample and an English-speaking sample) to be compared to establish cross-cultural validity [26]. There are methods for examining the cross-cultural validity both during the development of a PRO measure (e.g., translatability review) and for translation after a PRO measure has been developed in a language, usually English [41] (e.g., linguistic validation). For example, the PROMIS instruments underwent a translatability review in the development of the subjective well-being item pool [42]. Items were modified or removed if the content or sentence structure could not easily be translated into Spanish or German [42]. The practice of translatability assessment is still being refined [41]. Guidelines for the translation of PRO measures have been published by the MAPI Research Institute [43] and the PRO Consortium [44]. Clinicians may consider the original language used to develop a PRO measure and the methods by which other language translations were developed when choosing a PRO measure for their work.

Some limitations of the current study should be noted. We chose to focus our review on the most commonly used existing PRO measures and did not include every PRO measure that is used with pediatric cancer patients and survivors. There are other infrequently used PRO measures that may capture content domains different from the PRO measures reported here. Another limitation is that we only conducted our literature search in PubMed, a database indexing key clinical journals for pediatric oncologists and other clinicians, because we wanted to focus on PRO measures that would be used by these healthcare professionals. PubMed also includes PubMed Central (or PMC), which is a free digital repository that archives open access full-text articles published in biomedical and life sciences journals. Our review was not a systematic review, so it was not our intention to search for articles from all databases. In addition, we limited our search to articles and measures that were available in English, and we excluded articles published only in abstract form and those from grey literature. If we had included non-English and grey literature articles, we may have reviewed a broader scope of literature, but would have had difficulty finding high-quality articles and summarizing our findings [45].

There are many existing generic and cancer-specific measures used with pediatric cancer patients and survivors to assess PROs. However, these measures are varied in their content coverage across physical, psychological, and social domains and limited by their scoring methods and reported measurement properties. As such, there is no single gold standard PRO measure for this population. One set of PRO measures that is most comprehensive is the PROMIS [46]. This generic measure is useful for comparing outcomes of childhood cancer patients with different diagnoses to healthy children or children with other diseases. However, the PROMIS may not be sensitive to detecting changes of PRO scores over time that correspond to changes in underling health conditions (i.e., lacking in evidence of responsiveness). There is a need for a PRO measure that has both generic items for cross-disease comparison and cancer-specific items that are sensitive to detect differences among various cancer diagnoses and treatment modalities. To appropriately use PRO measures in pediatric oncology, clinicians and researchers should consider key issues when choosing a PRO measure to use: what is the purpose of using the PRO measure—for classification, prediction, and/or communication with patients? It is also important for clinicians to consider the measurement properties of a PRO measure that support its use in their cancer patient/survivor population for their purpose. Is this measure going to be used over the longitudinal course of a patient’s cancer journey? Is it going to be used as a screening measure? Is it going to be used in a group with a specific diagnosis? Is it going to be used in a multinational group of people who speak different languages? These questions will guide clinicians and researchers to choose the most suitable PRO measures that will best answer their specific questions and meet their specific needs. In the provided tables, we have summarized the characteristics (see Supplementary Table S1), content coverage (see Table 2 and Table 3), psychometric properties (see Table 4 and Supplementary Table S2), and language translations (see Supplementary Table S3) of each PRO measure. These summary tables can be used as references for clinicians and researchers to choose a PRO measure that is appropriate for their purpose and population. For example, a researcher may want to include a short, generic PRO measure of symptoms and emotional distress that is available in multiple languages. The tables reveal that the BDI, HUI, PedsQL-Core, and PROMIS all fit these criteria. From this narrowed down list, clinicians and researchers could examine the measurement properties and additional information about each measure and decide which to use in their clinical care or research.

Future research should focus on improving the clinical validity of existing PRO measures and clinical implementation of PRO measures. Before implementation, psychometric properties of the PRO measures must be considered to ensure that PRO measures are appropriately assessing the intended construct in childhood cancer patients. PRO measure implementation in clinical care is beneficial for patient-provider communication, focusing care on issues important to the patient, and providing needed referrals [47,48,49]. To assist in implementation, a decision tool could be made in future research that addresses the above questions and guides clinicians to choose the most suitable PRO measure for a particular pediatric cancer patient or survivor population and that focuses on the most relevant PRO content and contains appropriate measurement properties. The implementation of PRO measures into clinical care can provide clinicians with evidence of the patient experience that can be used to improve the patient’s HRQOL. Collecting patient-reported symptoms is especially meaningful and clinically actionable, which has shown HRQOL improvement for adult cancer patients [50]. However, the implementation of this strategy is still uncommon in pediatric cancer care and clinical trials [51]. Additionally, PROs related to the patient’s family and relationships (e.g., parenting behavior, family cohesion) are associated with symptom burden and HRQOL in young childhood cancer survivors [52]. This suggests that assessing social domains of PROs may also provide information to clinicians that can be addressed in clinical care to improve HRQOL. Therefore, it is important to conduct routine PRO assessment and monitoring in pediatric cancer care to facilitate clinical decision-making and improve quality of care for these patients.

## Figures and Tables

**Table 1:** Abbreviations of PRO assessment tool names.

Abbreviation	Full Name
**Generic measures**	
BDI	Beck Depression Inventory
CDI	Children’s Depression Inventory
CHIP	Child Health and Illness Profile
CHQ	Child Health Questionnaire
DCGM	DISABKIDS Chronic Generic Module
FPSR	Faces Pain Scale-Revised
HADS	Hospital Anxiety and Depression Scale
HUI	Health Utilities Index
KIDSCREEN	KIDSCREEN
KINDL	KINDL
PedsQL-Core	Pediatric Quality of Life Inventory Core Module
PedsQL-MFM	Pediatric Quality of Life Inventory Multidimensional Fatigue Module
PPS	Play Performance Scale
PROMIS	Patient-Reported Measurement Information System
RCMAS	Revised Children’s Manifest Anxiety Scale
STAIC	State-Trait Anxiety Inventory for Children
TNO-AZL	TNO AZL Quality of Life
**Cancer-specific measures**	
BASES	Behavioral Affective and Somatic Experiences Scale
ChIMES	Children’s International Mucositis Evaluation Scale
FS	Fatigue Scale
MMQL	Minneapolis-Manchester Quality of Life
MSAS	Memorial Symptom Assessment Scale
OMDQ	Oral Mucositis Daily Questionnaire
Pain Squad App	Pain Squad Application
PCQL-32	Pediatric Cancer Quality of Life Inventory-32
PEDQOL	Quality of Life in Children and Adolescents with Cancer
PedsFACT-Brs	Pediatric Functional Assessment of Cancer Therapy-Childhood Brain Tumor Survivors
PedsQL-Brain Tumor	Pediatric Quality of Life Inventory Brain Tumor Module
PedsQL-Cancer	Pediatric Quality of Life Inventory Cancer Module
PeNAT	Pediatric Nausea Assessment Tool
POQOLS	Pediatric Oncology Quality of Life Scale
SSPedi	Symptom Screening in Pediatrics Tool
TRSC-C	Therapy-Related Symptom Checklist-Children

**Table 2:** Content coverage for generic PRO measures used in pediatric cancer populations by major domains and subdomains.

	BDI	CDI	CHIP	CHQ	DCGM	FPSR	HADS	HUI	KIDSCREEN	KINDL	PedsQL-Core	PedsQL-MFM	PPS	PROMIS	RCMAS	STAIC	TNO-AZL
**Physical health**																	
Function ^a^	X		X	X	X			X	X	X				X			X
Symptoms ^b^	X		X	X		X		X			X	X		X			X
Functional independence ^c^			X		X			X	X	X	X						X
**Psychological health/functioning**																	
Body image ^d^	X										X						
Self-esteem ^e^	X	X	X	X					X	X							
Positive psychological function ^f^	X		X							X				X			X
Behavior ^g^	X		X	X											X		X
Cognitive ^h^	X		X					X			X	X		X	X		X
Emotional distress ^i^	X	X	X	X	X		X	X	X		X			X	X	X	X
**Social health**																	
Relationship ^j^	X	X	X	X					X	X	X			X			
Function ^k^			X	X	X						X						X
School functioning/social behavior ^l^			X	X					X								
**General health perception ^m^**				X										X			
**Cancer diagnosis/treatment ^n^**																	
**Other**									X ^o^				X ^p^				

Bolded domains are broad content categories and non-bolded domains are subcategories within those broad domains. Content domain categories based on previous reviews [20,30]. Presence of reported content area indicated with an X. See Table 1 for full names of PRO assessment tools. See Supplementary Table S4 for references for each PRO measure; ^a^ Activity, acute major disorders, acute minor disorders, ambulation, dexterity, effect of pain on sleeping, hearing, limitations of activity, long-term medical, long-term surgical, loss of libido, mobility, motor functioning, movement and balance, physical activity, physical function and role restriction, physical function-mobility, physical function-upper extremity, physical functioning, physical limitation, physical stress experience, physical well-being, problems/limitations concerning general physical functioning/complaints, recurrent disorders, role/social-physical, sleep, sleep disturbance, speech, strength impact, vision; ^b^ Abdominal pain, affective, amount of diarrhea, amount of mouth and throat pain, amount of pain medication received, appetite, appetite loss, asthma impact, bleeding/hematuria, bodily pain, bone marrow toxicity/neuropathy, change in health, comfort, constipation, diarrhea, eating more/less, fatigue, fertility, headache, ineffectiveness, lack of energy, lung problems, malaise, mouth sores, mucositis, nausea, neurotoxicities, not able to function, oropharyngeal, pain, pain intensity, pain quality-affective, pain quality-sensory, pain-behavior, pain-interference, physical discomfort, physical severity, physical symptoms, psychosocial/central nervous system, respiratory/other, skin problems, sleep-related disturbance, sleep-related impairment, sleep/rest fatigue, somatic distress, somatic preoccupation, symptom severity: nutrition related, throwing up, tingly hands/feet, tired, weight loss; ^c^ Autonomy, drooling-pooling of saliva, effect on drinking, effect on eating, effect on talking, effect on drinking, effect on swallowing, home safety and health, independence, individual risks, functional status in the activities of daily living, problems/limitations concerning independent daily functioning, self-care; ^d^ Appearance: presence of ulcers, body image, changes to how face/body look, perceived physical appearance; ^e^ body image (serves as an indicator of self-esteem), self-esteem, self-perception, sense of failure, self-hate, self accusations, self punitive wishes; ^f^ Engagement-persistence, life satisfaction, liveliness, meaning and purpose, positive affect, satisfaction with health, engagement-curiosity, lack of satisfaction, outlook on life, positive mood, satisfaction; ^g^ Aggressive, complaining/demanding, cooperation/compliance (oral medications, mouth care, bath/sitz bath, Hickman line care, vital signs/physical examination, eating/drinking, exercise/physical therapy, overall cooperation), regressed behavior, self-stimulatory behavior, talkative/engaging, crying spells, defensiveness, general behavior, problem behavior, risk avoidance; ^h^ Cognitive fatigue, cognitive functioning, cognitive problems, communication, indecisiveness, problem-solving, problems/limitations concerning cognitive functioning and school performances, problems with thinking/memory, self-regulation—flexibility, self-regulation—frustration tolerance, sensation, cognition; ^i^ Angry/irritable, confused/disoriented, depression, emotional distress-anxiety, emotional distress-depressive symptoms, fearful/anxious, guilty feeling, irritability, moods & emotions, pessimism, psychological stress experiences, psychosocial disorder, restless/agitated, role/social-emotional/behavioral, sad/subdued, sense of punishment, social anxiety/fear/concentration, the occurrence of negative moods, the occurrence of positive moods, trait anxiety, withdrawn, worry/oversensitivity, altered mood, anxiety, cheerful/friendly, emotional discomfort, emotional distress, emotional distress-anger, emotional functioning, emotional well-being, emotional well-being and illness experiences, feelings (disappointed/sad, scared/worried, moody/angry), mental health, mood, negative mood, physiological anxiety, Psychologic Functioning: This scale encompasses emotional functioning and includes items that involve emotional distress and worry, psychological functioning (emotional distress), psychological functioning (encompasses emotional functioning), psychological severity, frequency, and distress, psychological well-being, state anxiety, worry; ^j^ Bullying, community, family, family dynamics, family involvement, friends, interactions (quality of patient-parent interaction, quality of patient-staff interaction, quality of parent-staff interaction, parent in room), interpersonal problems, intimate relations, parent relation & home life, parental emotional impact, parental time impact, peer, peer influences, physician/nurse communication, resilience, significant others, social, social withdrawal, social/family well-being, teachers; ^k^ Achievement, anhedonia, family activities, family cohesion, interpersonal problems related to cancer and biomedical treatment, problems/limitations in social contacts with parents and peers, social exclusion, social functioning, social functioning (inter-personal functioning in peer relations), social functioning friends, social inclusion, threats to achievement; ^l^ Academic performance, school environment, work performance; ^m^ General health perceptions, global distress. global health; ^n^ Brain tumor survivor-specific concerns, disease and treatment-related symptoms, disease specific modules, procedural anxiety, reaction to current medical treatment, treatment, treatment anxiety, worry (about cancer and its treatment). ^o^ Financial resources; ^p^ Play for functional status.

**Table 3:** Content coverage for generic PRO measures used in pediatric cancer populations by major domains and subdomains.

	BASES	ChIMES	FS	MMQL	MSAS	OMDQ	Pain Squad App	PCQL-32	PEDQOL	PedsFACT-Brs	PedsQL-Brain Tumor	PedsQL-Cancer	PeNAT	POQOLS	SSPedi	TRSC-C
**Physical health**																
Function ^a^	X	X	X						X	X	X			X		
Symptoms ^b^	X	X	X		X	X	X				X	X	X		X	X
Functional independence ^c^				X				X	X							
**Psychological health/functioning**																
Body image ^d^		X		X					X			X			X	
Self-esteem ^e^																
Positive psychological function ^f^	X			X												
Behavior ^g^	X															
Cognitive ^h^				X				X	X		X	X			X	
Emotional distress ^i^	X		X	X	X			X	X	X	X	X		X	X	
**Social health**																
Relationship ^j^	X			X						X						
Function ^k^				X				X	X							
School functioning/social behavior ^l^																
**General health perception ^m^**					X											
**Cancer diagnosis/treatment ^n^**								X		X	X	X		X		
**Other**																

Bolded domains are broad content categories and non-bolded domains are subcategories within those broad domains. Content domain categories based on previous reviews [20,30]. Presence of reported content area indicated with an X. See Table 1 for full names of PRO assessment tools. See Supplementary Table S4 for references for each PRO measure; ^a^ Activity, acute major disorders, acute minor disorders, ambulation, dexterity, effect of pain on sleeping, hearing, limitations of activity, long-term medical, long-term surgical, loss of libido, mobility, motor functioning, movement and balance, physical activity, physical function and role restriction, physical function-mobility, physical function-upper extremity, physical functioning, physical limitation, physical stress experience, physical well-being, problems/limitations concerning general physical functioning/complaints, recurrent disorders, role/social-physical, sleep, sleep disturbance, speech, strength impact, vision; ^b^ Abdominal pain, affective, amount of diarrhea, amount of mouth and throat pain, amount of pain medication received, appetite, appetite loss, asthma impact, bleeding/hematuria, bodily pain, bone marrow toxicity/neuropathy, change in health, comfort, constipation, diarrhea, eating more/less, fatigue, fertility, headache, ineffectiveness, lack of energy, lung problems, malaise, mouth sores, mucositis, nausea, neurotoxicities, not able to function, oropharyngeal, pain, pain intensity, pain quality-affective, pain quality-sensory, pain-behavior, pain-interference, physical discomfort, physical severity, physical symptoms, psychosocial/central nervous system, respiratory/other, skin problems, sleep-related disturbance, sleep-related impairment, sleep/rest fatigue, somatic distress, somatic preoccupation, symptom severity: nutrition related, throwing up, tingly hands/feet, tired, weight loss; ^c^ Autonomy, drooling-pooling of saliva, effect on drinking, effect on eating, effect on talking, effect on drinking, effect on swallowing, home safety and health, independence, individual risks, functional status in the activities of daily living, problems/limitations concerning independent daily functioning, self-care; ^d^ Appearance: presence of ulcers, body image, changes to how face/body look, perceived physical appearance; ^e^ body image (serves as an indicator of self-esteem), self-esteem, self-perception, sense of failure, self-hate, self-accusations, self-punitive wishes; ^f^ Engagement-persistence, life satisfaction, liveliness, meaning and purpose, positive affect, satisfaction with health, engagement-curiosity, lack of satisfaction, outlook on life, positive mood, satisfaction; ^g^ Aggressive, complaining/demanding, cooperation/compliance (oral medications, mouth care, bath/sitz bath, Hickman line care, vital signs/physical examination, eating/drinking, exercise/physical therapy, overall cooperation), regressed behavior, self-stimulatory behavior, talkative/engaging, crying spells, defensiveness, general behavior, problem behavior, risk avoidance; ^h^ Cognitive fatigue, cognitive functioning, cognitive problems, communication, indecisiveness, problem-solving, problems/limitations concerning cognitive functioning and school performances, problems with thinking/memory, self-regulation—flexibility, self-regulation—frustration tolerance, sensation, cognition; ^i^ Angry/irritable, confused/disoriented, depression, emotional distress-anxiety, emotional distress-depressive symptoms, fearful/anxious, guilty feeling, irritability, moods & emotions, pessimism, psychological stress experiences, psychosocial disorder, restless/agitated, role/social-emotional/behavioral, sad/subdued, sense of punishment, social anxiety/fear/concentration, the occurrence of negative moods, the occurrence of positive moods, trait anxiety, withdrawn, worry/oversensitivity, altered mood, anxiety, cheerful/friendly, emotional discomfort, emotional distress, emotional distress-anger, emotional functioning, emotional well-being, emotional well-being and illness experiences, feelings (disappointed/sad, scared/worried, moody/angry), mental health, mood, negative mood, physiological anxiety, Psychologic Functioning: This scale encompasses emotional functioning and includes items that involve emotional distress and worry, psychological functioning (emotional distress), psychological functioning (encompasses emotional functioning), psychological severity, frequency, and distress, psychological well-being, state anxiety, worry; ^j^ Bullying, community, family, family dynamics, family involvement, friends, interactions (quality of patient-parent interaction, quality of patient-staff interaction, quality of parent-staff interaction, parent in room), interpersonal problems, intimate relations, parent relation & home life, parental emotional impact, parental time impact, peer, peer influences, physician/nurse communication, resilience, significant others, social, social withdrawal, social/family well-being, teachers; ^k^ Achievement, anhedonia, family activities, family cohesion, interpersonal problems related to cancer and biomedical treatment, problems/limitations in social contacts with parents and peers, social exclusion, social functioning, social functioning (inter-personal functioning in peer relations), social functioning friends, social inclusion, threats to achievement; ^l^ Academic performance, school environment, work performance; ^m^ General health perceptions, global distress. global health; ^n^ Brain tumor survivor-specific concerns, disease and treatment-related symptoms, disease specific modules, procedural anxiety, reaction to current medical treatment, treatment, treatment anxiety, worry (about cancer and its treatment).

**Table 4:** Presence of measurement properties tested in PRO measures for pediatric cancer populations.

PRO Measure	Structural Validity	Internal Consistency	Reliability	Construct Validity/Known-Group Validity	Cross-Cultural Validity/ Measurement Invariance	Criterion Validity	Responsiveness	Predictive Validity	Cut Points/MIDs	Response Shift	Score Calculation
**Generic**											
BDI		PedG, PedO		PedG		PedO		PedG	PedG, PedO	PedG	BDI-II: sum score, BDI-Y: T scores (standardized based on age and gender)
CDI	PedG	PedO		PedG, PedO							Sum score
CHIP	PedG	PedG	PedG	PedG	PedG						Standardized scores with mean of 20 and standard deviation of 5 or mean of 50 and standard deviation of 10
CHQ	PedO	PedO	PedO	PedO			PedO				Sum score, then converted to 0–100 scale
DCGM		PedG, PedO		PedG, PedO	PedG						Sum score, then converted to 0–100 scale
FPSR			PedG	PedG, PedO	PedG						Single item score 0–10
HADS	AduO	AduO			AduO		PedO				Sum score
HUI			PedO	PedO			PedO				Utility function from preference scores based on Neumann-Morganstern utility theory, VAS, then SG, 0 (dead) to 1 (perfect health)
KIDSCREEN	PedG, PedO	PedG	PedG PedO	PedG, PedO					PedO	PedG	T-values based on Rasch person parameters
KINDL	PedO	PedO		PedO		PedO	PedO				Sum score, then converted to 0–100 scale
PedsQL-Core	PedG	PedO	PedO	PedO	PedO		PedO		PedG	PedO	Mean score for psychosocial health scale, physical health scale, and total scores; transform scores to 0–100 scale
PedsQL-MFM		PedO	PedO	PedO							Mean score for general fatigue scale, sleep/rest fatigue scale, cognitive fatigue scale, and total scores; transform scores to 0–100 scale
PPS			PedO	PedO	PedO						Single item, 0–100 in increments of 10
PROMIS	PedO	PedO	PedO	PedO	PedO		PedO	PedO	PedO		T-score with mean of 50 and standard deviation of 10). In most cases 50 equals the mean in the U.S. general population.
RCMAS	PedO	PedO	PedG	PedO	PedG, PedO						Sum scores
STAIC		PedO		PedO				PedO			Sum scores
TNO-AZL		PedO		PedO	PedO		PedO				Sum score then converted to 0–100 score, TACQOL: no total score, just summed subdomain scores
**Cancer-specific**											
BASES		PedO	PedO	PedO	PedO		PedO				Sum scores
ChIMES		PedO	PedO	PedO	PedO		PedO				Sum scores
FS	PedO	PedO	PedO	PedO	PedO		PedO	PedO			Sum scores
MMQL	PedO	PedO	PedO	PedO			PedO				Mean scores
MSAS	AduO, PedO	PedO	PedO	AduO, PedO							Mean score
OMDQ		PedO	PedO	PedO							Total score has not been validated—examine items separately
Pain Squad App		PedO		PedO				PedO			Total score has not been validated—examine items separately
PCQL-32		PedO	PedO	PedO							Scores converted to z scores then T scores with mean of 50 and standard deviation of 10
PEDQOL		PedO	PedO	PedO, PedG							Answers to positive questions with Often and always were rated positive and answers to negative questions with often and always were rated negative. Evaluation of results was done as percentage of negative and positive answers to each single item.
PedsFACT-Brs	PedO	PedO	PedO	PedO	PedO						Sum, multiply by number of items in subscale, divide by number of items answered = symptom index score
PedsQL-Brain Tumor	PedO	PedO	PedO	PedO							Mean score for cognitive problems scale, pain and hurt scale, movement and balance scale, procedural anxiety scale, nausea scale, worry scale, and total scores; transform scores to 0–100 scale
PedsQL-Cancer	PedO	PedO	PedO	PedO			PedO				Mean score for pain and hurt scale, nausea scale, procedural anxiety scale, treatment anxiety scale, worry scale, cognitive problems scale, perceived physical appearance scale, communication scale, and total scores; transform scores to 0–100 scale
PeNAT			PedO	PedO			PedO				1–4 from single item
POQOLS	PedO	PedO	PedO	PedO	PedO		PedO				Sum scores
SSPedi			PedO	PedO							Sum scores
TRSC-C	PedO	PedO	PedO	PedO	PedO						Sum score

Psychometric property categories based on COSMIN guidelines [26,27]. PedG = Pediatric general (non-oncology) sample, PedO = Pediatric oncology patient/survivor sample, AduO = Adult oncology sample. See Table 1 for full names of PRO assessment tools. See Supplementary Table S4 for references for each PRO measure.

## Supplementary Materials

The following supporting information can be downloaded at: https://www.mdpi.com/article/10.3390/children9101497/s1, Supplementary Table S1. Characteristics of PRO measures used in pediatric cancer populations; Supplementary Table S2. Specific research reporting the measurement properties of PRO measures for pediatric cancer populations; Supplementary Table S3. Available language translations of PRO measures for pediatric cancer populations; Supplementary Table S4. Key references for PRO measures included in the present review study for pediatric cancer populations.
