# Supplementary material for: A Review of Patient-Reported Outcome Measures in Childhood Cancer
Source: Children (Basel). 2022 Sep 30;9(10):1497. doi: 10.3390/children9101497 (PMC9601091; doi:10.3390/children9101497)
Supplement: Supplementary file 1 [file children-09-01497-s001.zip › children-1893842-supplementary.pdf]

Supplementary Materials

Supplementary Table S1. Characteristics of PRO measures used in pediatric cancer populations.

PRO measure	Forms	Developer (year)	PRO type	Self-proxy-report (age range)	Recall period	Author of first application in pediatric cancer (year)	Cancer diagnosis or treatment/ treatment status (only first application regardless of form – can add first study from additional forms if desired)	Measurement theory used to create PRO measure	Number of items	Type of response scale	License free
**Generic**											
BDI	BDI,<br>BDI-II,<br>BDI-Short Form (BDI-SF),<br>BDI-Youth (BDI-Y),<br>BDI-Y-SF (including 6/8/11-item SF)	BDI: Beck et al. (1961)<br>BDI-II: Beck, Steer, & Brown (1996)<br>BDI-SF: Beck & Beck (1972)<br>BDI-Y: Beck, Beck, Jolly, & Steer (2005)<br>BDI-Y-SF: Blackmon JE (2015)	Health profile	Self (13-80 yrs)	BDI: current status, BDI-II: two weeks	Tebbi, Bromberg, & Mallon (1988)[a]	Sample (age): Cancer patients (adolescence)<br>n = 30<br>Language (country): English (US)<br>Diagnoses: Not reported<br>Treatment: Not reported	Clinically derived items. Beck made systematic observations and records of depressed patients and used these to create attitude and symptom items.	BDI: 21,<br>BDI-SF: 13,<br>BDI-Y: 20	Likert-type from 0 to 3, varied response options	No – available at https://www.pearsonassessments.com/
CDI		Kovacs M (1985)	Health profile	Self (7-17 yrs)	two weeks	Greenberg (1989)	Sample (age): Cancer survivors at least two years post treatment (8-16 yrs)<br>n = 135 patient-mother pairs, 92 healthy control-mother pairs<br>Language (country): English (US)<br>Diagnoses: 35% leukemia, 7% lymphoma, 46% solid tumor, 12% other [b]<br>Treatment: 30% chemotherapy and radiation and surgery, 40% chemotherapy and radiation, 18% chemotherapy and surgery, 4% radiation and surgery, 8% single-modality therapy	Unknown	27	Respondents choose one of three statements that describes feelings	No – available at https://www.pearsonassessments.com/

CHIP	CHIP, CHIP-Child Edition/Child Report Form (CHIP-CE/CRF), CHIP-Child Edition/Parent Report Form (CHIP-CE/PRF), CHIP-Adolescent Edition (CHIP-AE)	CHIP: Starfield B (1993) CHIP-CE/CRF: Riley (2004a) CHIP-CE/PRF: Riley (2004b) CHIP-AE: Starfield (1995)	Health profile	CHIP: Self (11-17 yrs) CHIP-CE/CRF: Self (6-11 yrs) CHIP-CE/PRF: Proxy (6-11 yrs) CHIP-AE: Self (11-17 yrs)	CHIP-CE/CRF: 4 weeks, CHIP-AE: varies across items from current status to 1 year	Klosky (2012)	Sample (age): Cancer survivors at least 5 years post Diagnoses (14-20 yrs)<br>n = 307 survivors, 97 healthy siblings<br>Language (country): English (US)<br>Diagnoses: 31% leukemia, 1% lymphoma, 13% CNS tumor, 55% solid tumor<br>Treatment: 32% chemotherapy and radiation, 47% chemotherapy only, 3% radiation only, 13% surgery only, 1% no chemotherapy or radiation or surgery, 3% unknown<br>Additional treatment: 63% no CNS treatment, 37% any CNS treatment	Expert agreement, Classical Test Theory (CTT)	CHIP-CE/CRF: 45, CHIP-CE/PRF: 76, CHIP-AE: 107 plus 46 optional disease/injury specific items	CHIP-CE/CRF: Likert-type from 1 (Never) to 5 (Always), visual scale	No – available at https://www.ideaconnection.com/
CHQ	CHQ-Parent Form 50 items (CHQ-PF50), CHQ-PF28, CHQ-Child Form 87 items (CHQ-CF87), CHQ-CF45	CHQ-PF50, CHQ-PF28, CHQ-CF87: Landgraf (1999) CHQ-CF45: Landgraf (2018)	Health profile	CHQ-PF: parent proxy CHQ-CF: self (5-18 yrs)	Most items are 4 weeks	Sawyer (1999)	Sample (age): Adolescents with cancer (10-18 yrs)<br>n = 70 adolescent-caregiver pairs<br>Language (country): English (Australia)<br>Diagnoses: 50% leukemia, 16% lymphoma, 3% CNS tumor, 13% solid tumor, 19% other [b]<br>Treatment: 34% on treatment, 66% off therapy	Unknown – Need license to access development/theory	CHQ-PF50: 50, CHQ-PF28: 28, CHQ-CF87: 87, CHQ-CF45: 45	Likert-type from 1 to 4, 1 to 5, or 1 to 6, varied response options	No – available at https://www.healthactchq.com/survey/chq
DCGM	DCGM-12, DCGM-37, Smileys modules	The DISABKIDS Group Europe (2006)	Health profile	Self and proxy (8-18 yrs), Smiley versions (4-7 yrs)	4 weeks or 1 week	af Sandeberg (2008)	Sample (age): Children newly diagnosed with cancer (7-16 yrs)<br>n = 101<br>Language (country): Swedish (Sweden)<br>Diagnoses: 37% leukemia, 21% lymphoma, 16% CNS tumor, 23% solid tumor, 3% other [b]<br>Treatment:	Bottom up (patient-derived). Lit review, focus groups, item selection, translations, pilot study, field study, implementation study (Baars 2005)	DCGM-12: 12, DCGM-37: 37	Likert-type from 1 (never) to 5 (always)	No – must submit collaboration form to DISABKIDS group

							chemotherapy and/or radiation				
FPSR	FPS, FPSR	FPS: Bieri (1990)<br>FPSR: Hicks (2001)	Health profile	Self (4-16 yrs)	Immediate	FPSR: Spagrud (2008)	Sample (age): Children with cancer undergoing routine blood work (3-18 yrs)<br>n = 55 child-parent pairs<br>Language (country): English (Canada)<br>Diagnoses: 57% leukemia, 4% lymphoma, 6% CNS tumor, 18% solid tumor,15% other [c]<br>Treatment: Not reported	For FPS: used children's drawings of facial expressions of pain to create 5 sets of facial expressions then validated with children. For FPS-R: cross-modality matching methodology	1	0-10 visual scale	Yes – need to submit permission request to reproduce
HADS		Zigmond (1983)	Health profile	Self (17+ yrs)	1 week	Berard (1998)	Sample (age): Adolescents newly diagnosed with cancer (12-19 yrs)<br>n = 43<br>Language (country): English (US)<br>Diagnoses: 65% hematological,12% CNS tumor, 21% solid tumor, 2% other [b]<br>Treatment: Not reported	Expert agreement	14	Likert-type from 1 to 3, varied response options	No – available at https://www.gl-assessment.co.uk/assessments/products/hospital-anxiety-depression-scale/
HUI	HUI-2, HUI-3, 16 versions of HUI in English based on mode of administration, assessment viewpoint, duration of assessment period, language	Feeny (1996)	Health profile and health preference	Self (12+ yrs), self by interview (9+ yrs), proxy (5+ yrs, proxy by interview (5+ yrs)	Varies across items by 1 week, 2 weeks, 4 weeks, or usual (no specified recall period)	Barr (1997)	Sample (age): Children with ALL on active therapy (11 mo-14 yrs)<br>n = 18 child-parent pairs<br>Language (country): English (Canada)<br>Diagnoses: 100% leukemia<br>Treatment: 100% chemotherapy	Multi-attribute utility theory	Self-administered: 15, Interview-administered: 40	3, 4, 5, or 6 levels of each attribute.	No – available at http://www.healthutilities.com/
KIDSCREEN	KIDSCREEN-52, KIDSCREEN-27, KIDSCREEN-10	KIDSCREEN Group (2006)	Health profile	Self and proxy (8-18 yrs)	1 week	Engelen (2011)	Sample (age): Children as soon as finished treatment (M = 9.25 yrs, SD = 5.06; only those 8-18 yrs completed the KIDSCREEN-52)<br>n = 191<br>Language (country):	Literature reviews, expert consultation, focus groups, IRT, structural equation modelling (SEM)	KIDSCREEN-52: 52, KIDSCREEN-27: 27, KIDSCREEN-10: 10	Likert-type from 1 (never) to 5 (always)	No – must submit collaboration form to KIDSCREEN group https://www.kidscreen.org/englis

							Dutch (Netherlands)<br>Diagnoses: 36% leukemia, 12% lymphoma, 8% CNS tumor, 37% solid tumor, 7% other [b]<br>Treatment: 96% chemotherapy, 29% radiation, 53% surgery, 9% SCT				h/conditions-of-use/
KINDL	Kiddy-KINDL, Kid-KINDL, Kiddo KINDO	Bullinger (1994), revised by Ravens-Sieberer & Bullinger (1998a, 1998b)	Health profile	Self (Kiddy-KINDL: 4-6 yrs; Kid-KINDL: 7-13 yrs, Kiddo-KINDL: 14-17 yrs), proxy (Kiddy-KINDL for parents: 3-6 yrs, Kid-/Kiddo-KINDL: 7-17 yrs)	1 week	Benesch (2009)	Sample (age): Survivors at least 2 years post Diagnoses (9-49 yrs)<br>n = 31<br>Language (country): German (Austria)<br>Diagnoses: 100% CNS tumor<br>Treatment: 91% chemotherapy and radiation, 9% chemotherapy only	Derived from conceptual model	Most versions: 24,<br>Self report Kiddy-KINDL: 12,<br>Parent version of Kiddy-KINDL: additional 22 items,<br>Short form: 12	Likert-type from 1 (never) to 3 (very often) for self report Kiddy-KINDL, 1 (never) to 5 (all the time) for all other versions	Yes – must submit collaboration form at https://www.kindl.org/escrib/terms-of-usage/
PedsQL-Core	Standard and acute versions	Version 1.0: Varni (1999)<br>Version 4.0: Varni (2002)	Health profile	Version 1.0: self and proxy (8-12 yrs and 13-18 yrs)<br>Version 4.0: self (5+ yrs), proxy (2+ yrs)	Standard version: 1 month, acute version: 7 days	Varni (1999)	Sample (age): Pediatric cancer patients (8-18 yrs)<br>n = 291 patient-parent pairs<br>Language (country): English (US)<br>Diagnoses: 44% leukemia,15 % lymphoma, 11% CNS tumor, 4% solid tumor, 26% other [b]<br>Treatment: 44% on treatment, 56% off treatment	Derived from the PCQL; expert agreement based on respondent interviews; further refined based on alpha, item-total correlations, reading level	Version 1.0: 15,<br>Version 4.0: 23,<br>Parent report (ages 2-4): 21,<br>Short form: 15	Version 1.0: Likert-type from 0 (it is never a problem) to 3 (it is always a problem)<br>Version 4.0: Likert-type from 0 (Never) to 4 (Always) and 0 (not at all), 2 (sometimes), 4 (a lot)	Yes – available at https://pedsql.org/conditions.html
PedsQL-MFM		Varni (2002)	Health profile	Self (5-18 yrs), parent proxy (2-18 yrs)	Standard version: 1 month, acute version: 7 days	Varni (2002)	Sample (age): Families of children with cancer (2-18 yrs)<br>n = 220 children, 337 parents<br>Language (country): English (US)<br>Diagnoses: 50% leukemia, 9% lymphoma, 7% CNS tumor,6 % solid tumor, 28% other [b]<br>Treatment: 54% on	Expert agreement, literature review, focus groups and interviews, field testing	18	Likert-type from 0 (Never) to 4 (Always) and 0 (not at all), 2 (sometimes), 4 (a lot)	Yes – available at https://pedsql.org/conditions.html

							treatment, 18% off treatment in recent remission, 28% off treatment long term				
PPS		Lansky (1985)	Health profile	Proxy (0-15 yrs)	1 week	Lansky (1987)	Sample (age): Pediatric oncology inpatients and outpatients (1-16 yrs)<br>n = 98 patients, 29 patient siblings, 40 healthy controls<br>Language (country): English (US)<br>Diagnoses: 60% leukemia/lymphoma, 4% CNS tumor, 23% solid tumor, 13% other [b]<br>Treatment: 75% chemotherapy, 3% radiation, 20% off treatment	Expert agreement, based on Gesell Scales of child development as model of play patterns	1	0-100 in increments of 10. Each response is a description of play/activity. Parent chooses which represents child best	Yes for students, physicians, clinical practice, not-funded academic users; no for funded academic users, healthcare organizations, commercial users & IT companies – see https://eprovide.mapi-trust.org/instruments/play-performance-scale-for-children#contact_and_conditions_of_use
PROMIS		Walsh (2008), Irwin (2009)	Health profile	Self (8-17 yrs), proxy (1-5 yrs, 5-17 yrs)	Varies across items by 4 weeks, 7 days, or in general	Hinds (2013)	Sample (age): Cancer patients on treatment or in survivorship following treatment (8-17 yrs)<br>n = 200 patients, 189 parents<br>Language (country): English (US)<br>Diagnoses: 60% leukemia/ lymphoma, 11% CNS tumor, 29% solid tumor<br>Treatment: 46% on treatment, 54% off treatment	Literature searches, focus groups, cognitive interviews, CTT, IRT	Self-report: 602 in 24 banks/scale/ pools and 34 short forms. Parent-report (ages 1-5): 225 in 12 banks/scales/ pools and 16 short forms. Parent report (5-17): 520 in 23 banks/scales/ pools and 31 short forms	Likert-type from 1 to 5 with varied response options, pain intensity: 0-10	Yes – available at https://eprovide.mapi-trust.org/instruments/play-performance-scale-for-children#contact_and_conditions_of_use
RCMAS	Ages 7-12 Child & Parent Forms, Ages 13-18	CMAS: Castenada (1956) RCMAS: Reynolds	Health profile	Self (6-19 yrs)	Current status	von Essen (2000)	Sample (age): Children and adolescents with cancer (8-18 yrs)<br>n = 51	Expert agreement, factor analysis	CMAS: 53, RCMAS: 37, RCMAS-2: 49,	Y/N	No – available at https://www.wpspublish.com/rcmas-2-revised-childrens-

	Adolescent & Parent Forms, also known as What I Think and Feel (WITF), Short form	(1978) RCMAS-2: Reynolds (2008)					Language (country): Swedish (Sweden) Diagnoses: 39% leukemia, 20% lymphoma, 4% CNS tumor, 36% solid tumor Treatment: 31% on treatment (100% chemotherapy, 25% radiation, 44% surgery), 69% off treatment (91% chemotherapy, 29% radiation, 51% surgery)		RCMAS-SF: 10		manifest-anxiety-scale-second-edition
STAIC		Spielberger (1973)	Health profile	Self and proxy (upper elementary or junior high school aged children)	State: current status, Trait: how you usually feel	Moore (1997) [a]	Sample (age): Children with cancer (9-18 yrs) n = 74 child-mother pairs Language (country): English (US) Diagnoses: Not reported Treatment: Not reported	Expert agreement – need license to access development/theory	40	Likert-type from 1 (hardly ever) to 3 (often) for trait, 3 response options related to feelings (e.g. I feel 1. Very upset, 2. Upset, 3. Not upset) for state	No – available at https://www.mindgarden.com/146-state-trait-anxiety-inventory-for-children
TNO-AZL	TAPQOL (preschool), TACQOL (children), TAAQOL (adult)	TACQOL: Verrips (1999) TACQOL parent form: Vogels (1998) TAPQOL: Fekkes (2000)	Health profile	TAPQOL: proxy (6 months-6 yrs) TACQOL: proxy and self (6-15 yrs)	TAPQOL: 3 months, TACQOL: the past few weeks	Koopman (2005)	Sample (age): Cancer survivors 3 and 8 years after treatment (6–18 yrs at baseline) n = 20 patients, 1122 healthy controls at 3 year follow up, 272 healthy controls at 8 year follow up Language (country): Dutch (Netherlands) Diagnoses: 100% solid tumor Treatment: 95% chemotherapy and radiation and surgery, 5% chemotherapy and radiation	Expert agreement	TAPQOL: 43, TACQOL: 56, TACQOL parent: 55	TAPQOL: Likert-type from 0 (has a problem and feels bad) to 4 (no problem), TACQOL: Likert-type from 0 to 3	Yes – available at https://www.tno.nl/en/focus-areas/healthy-living/roadmaps/youth/questionnaires-to-measure-health-related-quality-of-life/
**Cancer-specific**											
BASES	BASES, BASES-P (parent), BASES-C (child)	Phipps (1994)	Health profile	Nurse report, parent report, child report (not specific –	Nurse report: 8- to 12-hr shift, Parent and child reports: past 24 hr	Phipps (1994)	Sample (age): Nurses and parents of pediatric oncology inpatients (2-20 yrs) n = Study 1: 17 nurses, Study 2: 27 nurses, 18 nurse-	Expert agreement	Nurse and parent report: 38, Self-report: 14	5 point Likert-type, varied response options	Unknown

				sample age 2-20 yrs in study)			parent pairs<br>Language (country): English (US)<br>Diagnoses: heterogeneous sample of diagnoses, mention of one patient with AML and one patient with CML,<br>Treatment: 100% bone marrow transplant				
ChIMES		Tomlinson (2009a)	Health profile	Self, parent (8-18 yrs)	Current status	Tomlinson (2009b)	Sample (age): Cancer patients (1 mo-18 yrs)<br>n = 40 child-parent pairs<br>Language (country): English (Canada)<br>Diagnoses: Not reported<br>Treatment: 90% chemotherapy, 18% radiation	Expert agreement	6	Visual scale from 0 to 5, Y/N	Yes – available at https://www.sungresearch.com/chimes-child-report
FS	FS-C (child), FS-P (parent), FS-A (adolescent), FS-S (staff)	Theory in Hinds (1999) Hockenberry (2003); revised by Hinds (2010) Adolescent: Hinds (2007); revised by Mandrell (2011)	Health profile	Self (7-12 yrs, 13-18 yrs), parent, staff	Varies across items by 1 week or 24 hours	Hockenberry (2003)	Sample (age): Children with cancer receiving chemotherapy (7-12 yrs)<br>n = 149 patients, 147 parents, 124 staff<br>Language (country): English (US)<br>Diagnoses: 64% leukemia, 10% lymphoma, 26% solid tumor<br>Treatment: 100% chemotherapy	Focus group interview data in qualitative study, expert agreement; Rasch in Hinds 2010 and Madrell 2011	Child-report: 14, Child-report revised: 10, Parent-report: 17, Staff-report: 9, Adolescent-report: 14, Adolescent-report revised: 13	Child: Y/N, Likert-type from 0 to 4, Parent: Likert-type from 0 to 4<br>Staff: Likert-type from 0 to 3<br>Adolescent: Likert-type from 0 to 4	Yes – FS-A available in Mandrell (2011); Other scales not publicly available
MMQL	MMQL-YF (youth form), MMQL-AF (adolescent form)	MMQL-YF: Bhatia (2004), MMQL-AF: Bhatia (2002)	Health profile	YF: interview (8-12 yrs) AF: self (13-20 yrs)	Unknown	Bhatia (2002)	Sample (age): Adolescents with cancer (13-20 yrs)<br>n = 110 patients on treatment, 158 patients off treatment, 129 healthy controls,<br>Language (country): English (US)<br>Diagnoses: 45% leukemia, 22% lymphoma, 6% CNS tumor, 21% solid tumor, 6% other [b]<br>Treatment: 41% on treatment, 59% off treatment	Focus groups, interviews, factor analysis to refine, item-total correlation	MMQL-YF: 32, MMQL-AF: 46	Likert-type with 4 or 5 response options	Yes – obtain permission from authors

MSAS	MSAS-SF (short form), MSAS 7-12 (ages 7-12), MSAS 10-18 (pMSAS, ages 10-18)	MSAS: Portenoy (1994)<br>MSAS-SF: Chang (2000)<br>MSAS 7-12: Collins (2002)<br>MSAS 10-18: Collins (2000)	Health profile	Self (7-12 yrs, 10-18 yrs)	1 week	Collins (2000)	Sample (age): Pediatric oncology inpatients and outpatients (10-18 yrs)<br>n = 160<br>Language (country): English (US)<br>Diagnoses: 21% leukemia, 16% lymphoma, 11% CNS tumor, 34% solid tumor, 18% other [d]<br>Treatment: 44% chemotherapy	Expert agreement; factor analysis<br>MSAS 10-18: simplified based on expert agreement and reading level	MSAS-GDI: 10,<br>MSAS-PHYS: 12,<br>MSAS-PSYCH: 6,<br>MSAS-SF: 32,<br>MSAS 10-18: 30	Likert-type with varied response options	Yes – MSAS-SF available at https://www.mids.org/content/memorial-symptom-assesment-scale-%E2%80%93-short-form-msas-sf<br>MSAS available in Portenoy (1994).<br>MSAS 10-18 available in Collins (2000).<br>MSAS 7-12 available in Collins (2002).
OMDQ		Stiff (2006)	Health profile	Self (18+ yrs) and interview when applicable; parent (1-12 yrs)	24 hours	Tomlinson (2011)	Sample (age): Parents of children with cancer (1-12 yrs)<br>n = 59<br>Language (country): English (US)<br>Diagnoses: 44% leukemia/ lymphoma, 12% CNS tumor, 15% solid tumor, 29% other [e]<br>Treatment: 27% chemotherapy, 73% SCT	Focus groups, interviews	6	Likert-type from 0 to 10, 0 to 4, or 0 to 3; Y/N; Tomlinson (2011) modified with visual scales for children	Yes – available at https://www.facit.org/measures/omdq
Pain Squad App	Pain Squad+	Pain Squad app: Stinson (2013)<br>Pain Squad+: Jibb (2017)	Health profile	Self (8-18 yrs)	Current status	Stinson (2013)	Stinson 2013<br>Sample (age): Adolescent inpatients and outpatients with cancer (9-18 yrs)<br>n = 47<br>Language (country): English (Canada)<br>Diagnoses: 47% leukemia, 11% lymphoma, 32% solid tumor, 11% other [b]<br>Treatment: Not reported	Expert agreement	20	Visual analog slider to rate dimensions of pain from 0-10, selectable body map of pain locations, multiple choice questions about pain characteristics, list of selectable words describing pain, free-text question	Yes – available in the Apple app store
PCQL-32		Varni (1998b)	Health profile	Self and parent (8-12 yrs, 13-18 yrs)	1 month	Varni (1998b)	Sample (age): Pediatric cancer patients (8-18 yrs)<br>n = 291 patient-parent pairs	interviews, expert agreement,	32	Likert-type from 0 (never a problem) to 4 (it is always a problem)	Unknown

							Language (country): English (US)<br>Diagnoses: 51% leukemia, 15% lymphoma, 11% CNS tumor, 19% solid tumor, 5% other [b]<br>Treatment: 45% on treatment (chemotherapy or radiation), 55% off treatment				
PEDQOL		Calaminus (2000)	Health profile	Self and proxy (5+ yrs)	Unknown	Calaminus (2000)	Sample (age): Cancer survivors 1-5 years off treatment (8-17 yrs)<br>n = 49 survivors, 62 healthy controls<br>Language (country): German (Germany)<br>Diagnoses: 33% leukemia,18 % lymphoma, 49% solid tumor<br>Treatment: 100% off treatment	Expert agreement	48	5 point Likert type from never to always	Unknown
PedsFACT-Brs	Child, parent, and adolescent forms	Lai (2007)	Health profile	Self (7-18 yrs), parent proxy	4 weeks	Lai (2007)	Sample (age): Cancer survivors at least 1 year post treatment (7-11 yrs)<br>n = 20 survivors, 20 caregivers, 12 experts<br>Language (country): English (US)<br>Diagnoses: 100% CNS tumor<br>Treatment: 20% chemotherapy, 25% radiation, 95% surgery	Interviews, expert agreement, readability, IRT, CTT	Child-report: 34, Parent-report: 37, Adolescent-report: 37	Likert-type from 0 (not at all) to 4 (very much)	Yes – available at https://www.facit.org/measures/peds-FACT-BR
PedsQL-Brain Tumor		Palmer (2007)	Health profile	Self (5-18 yrs), parent proxy (2-18 yrs)	Standard version: 1 month, acute version: 7 days	Palmer (2007)	Sample (age): Children with brain tumors on treatment and off treatment (2-18 yrs)<br>n = 51 patients, 99 parents<br>Language (country): English (US)<br>Diagnoses:100% CNS tumor<br>Treatment: 88% chemotherapy, 62%	Expert agreement, literature review, focus groups and interviews, field testing	Parent-report (2-4 yrs): 17, Parent- and self-report (5-7 yrs): 23, Parent- and child-report (8-12 yrs, 13-18 yrs): 24	Likert-type from 0 (never a problem) to 4 (almost always a problem)	Yes – available at https://pedsql.org/conditions.html

							radiation, 84% surgery, 5% SCT				
PedsQL-Cancer		Varni (2002)	Health profile	Self (5+ yrs), parent proxy (2+ yrs)	Standard version: 1 month, acute version: 7 days	Varni (2002)	Sample (age): Families of children with cancer (2-18 yrs) n = 220 children, 337 parents Language (country): English (US) Diagnoses: 50% leukemia, 9% lymphoma, 7% CNS tumor,6 % solid tumor, 28% other [b] Treatment: 54% on treatment, 18% off treatment in recent remission, 28% off treatment long term	Expert agreement, previous development of PedsQL	Parent-report (2-4 yrs): 25, Parent- and self-report (5-7 yrs): 26, Parent- and child-report (8-12 yrs, 13-18 yrs, 18-25 yrs, 26+ yrs): 27	Likert-type from 0 (Never) to 4 (Always) and 0 (not at all), 2 (sometimes), 4 (a lot)	Yes – available at https://pedsql.org/conditions.html
PeNAT		Dupuis (2006)	Health profile	Self (4+ yrs), interview administration	Varies across items by ever or immediate	Dupuis (2006)	Sample (age): Pediatric inpatients (4-18 yrs) n = 154 cancer patients, 23 inpatients without cancer Language (country): English (US) Diagnoses: Not reported Treatment: 100% chemotherapy	Expert agreement	1 visual scale, Ages 4-8: 9 interview questions, Ages 8+: 5 interview questions	Visual scale (4 faces), interview script	Yes – available in Dupuis (2006)
POQOLS		Goodwin (1994)	Health profile	Parent report (3-18 yrs)	2 weeks	Goodwin (1994)	Sample (age): Families of children with cancer (6-17 yrs) n = 40 parents, 20 children in Phase 1. 210 parents in Phase 3. 107 parents in Phase 4 Language (country): English (US) Diagnoses: Phase 3: 62% leukemia, 6% lymphoma, 15% solid tumor, 17% other [f]; Phase 4: 71% leukemia, 19% solid tumor, 10% other [f] Treatment: Not reported	Literature, interviews, factor analysis, item-total correlations	21	7 point Likert-type from never to very frequently	Yes – available in Goodwin (1994)
SSPedi	Mini-SSPedi: Ages 4-7, SSPedi: Ages 8-18, Proxy	Tomlinson (2014)	Health profile	Self (4-7 yrs, 8-18 yrs) proxy (8-18 yrs)	Varies across items by yesterday or today	O'Sullivan (2014)	Sample (age): Children with cancer (8-18 yrs) and their parents n = 30 children, 20	Nominal group technique (NGT) with patient and experts	15	Likert-type from 0 (not bothered at all) to 4	Yes – available at https://www.sungresearch.com/sspedi

	SSPedi: Proxy Form						parents<br>Language (country): English (Canada)<br>Diagnoses: Not reported<br>Treatment: 77% chemotherapy			(extremely bothered)	
TRSC-C		Williams (2006)	Health profile	Self (5-17 yrs)	Immediately after and since last treatment	Williams (2006)	Sample (age): Cancer patients (2-18 yrs) and their parents<br>n = 11<br>Language (country): English (US)<br>Diagnoses: 27% leukemia, 9% lymphoma, 9% CNS tumor, 55% solid tumor<br>Treatment: 100% chemotherapy, 0% radiation	Derived from TRSC (adults), 2012 used factor analysis, 2006 was uncalibrated	30 (plus write in options)	Likert-type from 0 (no symptom) to 4 (a whole lot)	Yes – need permission from authors

Measurement theory: Modern, non-modern, CTT, IRT, etc. See Table 1 for full names of pro assessment tools. See Supplementary Table S4 for references for each PRO measure; [a] no full text; [b] indicated as “other” in article; [c] other = nonmalignant diagnosis or diagnosis unavailable; [d] other = rare malignancies of childhood (carcinoma, etc.); [e] Other diagnoses include: aplastic anemia (*n* = 7), adrenoleukodystrophy (*n* = 2), hemophagocyticlymphohistiocytosis (*n* = 2), post-transplant lymphoproliferative disorder (*n* = 2), chronic granulomatous disease (*n* = 1), Langerhans cell histiocytosis (*n* = 1), Hurler’s syndrome (*n* = 1), and Fanconi’s anemia (*n* = 1); [f] Phase 3 other = variety of different diagnoses including Wilm’s Tumor, neuroblastoma, and Burkett’s lymphoma. Phase 4 other = various other diagnoses (e.g., Wilm’s tumor, Hodgkin’s disease, medulloblastoma).

Supplementary Table S2. Specific research reporting the measurement properties of PRO measures for pediatric cancer populations.

PRO measure	Structural validity	Internal consistency	Reliability	Construct validity/ known-group validity	Cross-cultural validity/ measurement invariance	Criterion validity	Responsiveness	Predictive validity	Cut points/ MIDs	Response shift	Score calculation
**Generic**											
BDI		Ambrosini (1991)<br>Blackmon (2015)<br>Blackmon (2017)		Ambrosini (1991)		Blackmon (2015)<br>Blackmon (2017)		Ambrosini (1991)	Ambrosini (1991)<br>Blackmon (2015)<br>Blackmon (2017)	Wu (2016)	BDI-II: sum score, BDI-Y: T scores (standardized based on age and gender)
CDI	Carey (1987)	Yoo (2010)		Carey (1987)<br>Durualp (2012)<br>Yoo (2010)							Sum score
CHIP	Riley (2004, CRF)<br>Riley (2004, PRF)<br>Starfield (1995)	Riley (2004, CRF)<br>Riley (2004, PRF)<br>Starfield (1995)	Riley (2004, CRF)<br>Riley (2004, PRF)<br>Starfield (1995)	Riley (2004, CRF)<br>Riley (2004, PRF)<br>Starfield (1995)	Riley (2004, CRF)<br>Riley (2004, PRF)<br>Starfield (1995)						Standardized scores with mean of 20 and standard deviation of 5 or mean of 50 and standard deviation of 10
CHQ	Hepner (2002)	Nixon Speechley (1999)	Waters (2003)	Bhatia (2002)<br>Nixon Speechley (1999)<br>Sung, Greenberg, Doyle, et al. (2003)<br>Sung, Greenberg, Young, et al. (2003)<br>Waters (2003)			Banks (2008)				Sum score, then converted to 0-100 scale
DCGM		Ravens-Sieberer (2007)<br>Santos (2016)		Ravens-Sieberer (2007)<br>Santos (2016)	Ravens-Sieberer (2007)						Sum score, then converted to 0-100 scale
FPSR			Bieri (1990)<br>Hicks (2001)	Baxter (2011)<br>Hicks (2001)	da Silva (2008)<br>Hicks (2001)						Single item score 0-10
HADS	Annunziata (2011)<br>Moorey (1991)	Moorey (1991)			Annunziata (2011)		Jorngarden (2007)				Sum score
HUI			Banks (2008)<br>Trudel (1998)	Nixon Speechley (1999)<br>Sung, Greenberg, Young, et al. (2003)<br>Trudel (1998)			Banks (2008)				Utility function from preference scores based on Neumann-Morganstern utility theory, VAS, then SG, 0 (dead) to 1 (perfect health)
KIDSCREEN	Jervaeus (2013)<br>Ravens-Sieberer (2008)	Ravens-Sieberer (2008)<br>Ravens-Sieberer (2010)	Jervaeus (2013)<br>Ravens-Sieberer (2008)<br>Ravens-Sieberer (2010)	Jervaeus (2013)<br>Ravens-Sieberer (2008)<br>Ravens-Sieberer (2010)					Jervaeus (2013)	Gillison (2008)	T-values based on Rasch person parameters
KINDL	Ergin (2015)	Ergin (2015)		Ergin (2015)		Ergin (2015)	Muller (2016)				Sum score, then converted to 0-100 scale
PedsQL-Core	Hoffman (2013)	Sato (2010)<br>Sato (2013)<br>Varni (1999)<br>Varni (2002)	Banks (2008)<br>Eiser (2003)<br>Varni (1999)	Eiser (2003)<br>Lau (2010)<br>Sato (2010)<br>Sato (2013)<br>Varni (1999)<br>Varni (2002)<br>Veldhuijzen Van Zanten (2017)	Eiser (2003)		Banks (2008)		Huang (2009)	Brinksma (2014)	Mean scores for subscales and total scores; transform scores to 0-100 scale
PedsQL-MFM		Varni (2002)	Tomlinson (2011)<br>Varni (2002)	Nunes (2017)<br>Palmer (2007)<br>Tomlinson (2011)<br>Varni (2002)<br>Veldhuijzen Van Zanten (2017)							Mean scores for subscales and total scores; transform scores to 0-100 scale

PPS			Lansky (1987)	Eiser (1997)<br>Klaassen (2010)<br>Lansky (1987)<br>Batra (2014)	Eiser (1997)						Single item, 0-100 in increments of 10
PROMIS	Liu (2015)<br>Liu (2019)<br>Thissen (2016)<br>Westmoreland (2018)	Hinds (2019)<br>Liu (2019)<br>Westmoreland (2018)	Liu (2015)<br>Thissen (2016)	Hinds (2013)<br>Liu (2019)<br>Westmoreland (2018)	Hinds (2013)<br>Liu (2015)		Hinds (2019)<br>Reeve (2018)	Hinds (2019)	Thissen (2016)		T-score with mean of 50 and standard deviation of 10). In most cases 50 equals the mean in the U.S. general population.
RCMAS	Wu (2016)	Santos (2016)<br>Wu (2016)	Reynolds (1978)	Santos (2016)<br>Wu (2016)	Reynolds (1978)<br>Wu (2016)						Sum scores
STAIC		Allen (1997)<br>Sato (2010)<br>Sato (2013)		Allen (1997)<br>Maurice-Stam (2011)<br>Sato (2010)<br>Sato (2013)				Sato (2013)			Sum scores
TNO-AZL		Koopman (2005)		Koopman (2005)<br>Landolt (2006)<br>Maurice-Stam (2008)	Landolt (2006)		Landolt (2006)<br>Maurice-Stam (2008)				Sum score then converted to 0-100 score, TACQOL: no total score, just summed subdomain scores
**Cancer-specific**											
BASES		Phipps (1994)<br>Phipps (1999)<br>Ullrich (2017)	Phipps (1994)<br>Phipps (1999)<br>Ullrich (2017)	Mehling (2012)<br>Phipps (1994)<br>Phipps (1999)<br>Rosipal (2013)	Phipps (1999)		Phipps (1999)				Sum scores
ChIMES		Jacobs (2013)<br>Paiva (2018)	Jacobs (2013)<br>Paiva (2018)	Jacobs (2013)<br>Khurana (2013)<br>Paiva (2018)	Paiva (2018)		Jacobs (2013)				Sum scores
FS	Chiang (2008)<br>Hinds (2007)<br>Hockenberry (2003)	Chiang (2008)<br>Hinds (2007)<br>Hockenberry (2003)	Hockenberry (2003)	Chiang (2008)<br>Hinds (2007)<br>Hockenberry (2003)	Hinds (2007)<br>Hockenberry (2003)		Hinds (2007)<br>Hockenberry (2003)	Chiang (2008)			Sum scores
MMQL	Bhatia (2004)<br>Hutchings (2007)<br>Koike (2014)	Bhatia (2002)<br>Bhatia (2004)<br>Einberg (2013)<br>Hutchings (2007)<br>Koike (2014)	Bhatia (2002)<br>Einberg (2013)<br>Hutchings (2007)<br>Koike (2014)	Bhatia (2002)<br>Bhatia (2004)<br>Hutchings (2007)<br>Koike (2014)<br>Shankar (2004)			Hutchings (2007)				Mean scores
MSAS	Collins (2000)<br>Portenoy (1994)	Collins (2000)<br>Li (2020)	Collins (2000)	Collins (2000)<br>Collins (2002)<br>Portenoy (1994)							Mean score
OMDQ		Cheng (2017)	Cheng (2017)<br>Tomlinson (2011)	Cheng (2017)<br>Jacobs (2013)<br>Paiva (2018)<br>Tomlinson (2011)							Total score has not been validated - examine items separately
Pain Squad App		Stinson (2015)		Stinson (2015)				Stinson (2015)			Total score has not been validated - examine items separately
PCQL-32		Seid (1999)<br>Varni (1998a)	Seid (1999)<br>Varni (1998b)	Seid (1999)<br>Varni (1998a)<br>Waters (2003)							Scores converted to z scores then T scores with mean of 50 and standard deviation of 10
PEDQOL		Calaminus (2000)	Heinks (2018)	Calaminus (2007)<br>Heinks (2018)<br>Müller (2001)<br>Seifert (2014)							Answers to positive questions with Often and always were rated positive and answers to negative questions with often and always were rated negative. Evaluation of

											results was done as percentage of negative and positive answers to each single item.
PedsFACT-Brs	Lai (2007)	Lai (2007)<br>Yoo, Kim (2011)<br>Yoo, Kim (2010)<br>Yoo, Ra (2011)	Yoo, Kim (2011)<br>Yoo, Kim (2010)<br>Yoo, Ra (2011)	Yoo, Kim (2011)<br>Yoo, Kim (2010)<br>Yoo, Ra (2010)<br>Yoo, Ra (2011)	Yoo, Ra (2011)						Sum, multiply by number of items in subscale, divide by number of items answered = symptom index score
PedsQL-Brain Tumor	Palmer (2007)<br>Sato (2010)<br>Sato (2013)	Sato (2010)<br>Sato (2013)	Palmer (2007)<br>Sato (2010)<br>Sato (2013)	Kuhlthau (2012)<br>Mandrell (2016)<br>Palmer (2007)<br>Sato (2010)<br>Sato (2013)							Mean scores for subscales and total scores; transform scores to 0-100 scale
PedsQL-Cancer	Lau (2010)<br>Santos (2016)<br>Tsuji (2011)	Lau (2010)<br>Felder-Puig (2004)<br>Santos (2016)<br>Scarpelli (2008)<br>Tsuji (2011)<br>Varni (2002)	Banks (2008)<br>Felder-Puig (2004)<br>Lau (2010)<br>Santos (2016)<br>Scarpelli (2008)<br>Tsuji (2011)<br>Varni (2002)	Banks (2008)<br>Felder-Puig (2004)<br>Lau (2010)<br>Santos (2016)<br>Scarpelli (2008)<br>Tsuji (2011)<br>Varni (2002)<br>Veldhuijzen Van Zanten (2017)			Banks (2008)				Mean scores for subscales and total scores; transform scores to 0-100 scale
PeNAT			Dupuis (2006)	Dupuis (2006)<br>Dupuis (2019)<br>Flank (2017)<br>Loves (2019)			Evans (2018)				1-4 from single item
POQOLS	Goodwin (1994)	Goodwin (1994)<br>Kazak (1996)	Goodwin (1994)<br>Kazak (1996)	Barrera (2006)<br>Goodwin (1994)<br>Kazak (1996)<br>Mounir (2007)	Barrera (2006)		Peeters (2009)				Sum scores
SSPedi			Dupuis (2018)<br>Hyslop (2018)	Dupuis (2018)<br>Hyslop (2018)<br>Loves (2019)							Sum scores
TRSC-C	Mansouri (2017)<br>Williams (2006)	Mansouri (2017)<br>Williams (2015)<br>Williams (2006)	Li (2013)<br>Mansouri (2017)	Li (2013)<br>Williams (2015)<br>Williams (2006)	Mansouri (2017)						Sum scores

Note. Psychometric property categories based on COSMIN guidelines [see References 26,27]. PedG = Pediatric general (non-oncology) sample, PedO = Pediatric oncology patient/survivor sample, AduO = Adult oncology sample. See Table 1 for full names of pro assessment tools. This table is not an exhaustive list of all articles with evidence of measurement properties, but rather a set of example articles. See Supplementary Table S4 for references for each PRO measure.

Supplementary Table S3. Available language translations of PRO measures for pediatric cancer populations.

PRO measure	Languages	Developer's website with access to translation of PRO measure
**Generic**		
BDI	English, Spanish	https://www.pearsonassessments.com/store/usassessments/en/Store/Professional-Assessments/Personality-%26-Biopsychosocial/Beck-Depression-Inventory-II/p/100000159.html#products
CDI	English	https://www.pearsonassessments.com/store/usassessments/en/Store/Professional-Assessments/Personality-%26-Biopsychosocial/Children%27s-Depression-Inventory-2/p/100000636.html?tab=product-details
CHIP	English (38 translations according to eprovide.mapi-trust.org)	https://www.eprovide.mapi-trust.org
CHQ	88 languages	https://www.healthactchq.com/translation/chq
DCGM	Dutch, English (UK), French, German, Greek, Swedish	https://eprovide.mapi-trust.org/search?form%5BsearchText%5D=DISABKIDS&form%5BfromAutocomplete%5D=0&form%5BsortByOrder%5D=&form%5Bpage%5D=1&form%5Bezxform_token%5D=lEYBnYpcJnX78Eyqf2Md7IXQBMjsqMBIoy44pUTCdwM
FPSR	69 languages (38 certified translation by Mapi)	https://www.iasp-pain.org/resources/faces-pain-scale-revised/#download
HADS	115 languages	https://www.gl-assessment.co.uk/assessments/products/hospital-anxiety-depression-scale/
HUI	31 languages	http://www.healthutilities.com/

KIDSCREEN	44 countries (38 languages)	https://www.kidscreen.org/english/language-versions/existing-language-versions/
KINDL	32 languages	https://www.kindl.org/english/language-versions/
PedsQL-Core	Core (standard): 89 countries, 78 languages.<br>Core (acute): 68 countries, 59 languages<br>Core SF15 (standard): 43 countries, 37 languages<br>Core SF15 (acute): English (USA)	https://eprovide.mapi-trust.org/instruments/pediatric-quality-of-life-inventory#languages
PedsQL-MFM	Standard: 59 countries, 50 languages<br>Acute: 56 countries, 49 languages	https://eprovide.mapi-trust.org/instruments/pediatric-quality-of-life-inventory#languages
PPS	English (USA)	https://eprovide.mapi-trust.org/instruments/play-performance-scale-for-children#languages
PROMIS	Pediatric: 43 languages, parent proxy: 41 languages	https://www.healthmeasures.net/explore-measurement-systems/promis/intro-to-promis/available-translations
RCMAS	French, Italian, Korean, Spanish, English	English: https://www.wpspublish.com/rcmas-2-revised-childrens-manifest-anxiety-scale-second-edition, French: https://www.pearsonclinical.fr/r-cmas-echelle-danxiete-manifeste-pour-enfants-revisee, Italian: https://www.giuntipsy.it/catalogo/test/rcmas-2, Korean: https://inpsyt.co.kr/main, Spanish: https://www.manualmoderno.com/
STAIC	27 languages	https://www.mindgarden.com/146-state-trait-anxiety-inventory-for-children#horizontalTab4
TNO-AZL	Dutch, English, German, French, Italian, Spanish, Vietnamese, Korean, Russian, Bulgarian	https://www.tno.nl/en/focus-areas/healthy-living/roadmaps/youth/questionnaires-to-measure-health-related-quality-of-life/

**Cancer-specific**		
BASES	English	Phipps (1994)
ChIMES	English	https://www.sungresearch.com/chimes-child-report
FS	English	Hockenberry (2003), Mandrell (2011), Hinds (2010)
MMQL	English	Bhatia (2002), Bhatia (2004)
MSAS	English	MSAS: Portenoy (1994)<br>MSAS-SF: Chang (2000)<br>MSAS 7-12: Collins (2002)<br>MSAS 10-18: Collins (2000)
OMDQ	English	https://www.facit.org/measures/omdq
Pain Squad App	English	Apple app store
PCQL-32	English	Varni (1998)
PEDQOL	German	Calaminus (2000)
PedsFACT-Brs	English, Finnish, Portuguese	https://www.facit.org/measure-languages/peds-FACT-Br-Languages
PedsQL-Brain Tumor	Standard: Mandarin Chinese (simplified; China), Polish (Poland), Portuguese (Portugal), English (USA)<br>Acute: Arabic (Egypt), English (USA), French (Canada, France), Japanese (Japan), Serbian (Latin; Serbia), Spanish (Uruguay, USA), Thai (Thailand)	https://eprovide.mapi-trust.org/instruments/pediatric-quality-of-life-inventory#languages

PedsQL-Cancer	Standard: 47 countries, 36 languages<br>Acute: English (USA), Italian (Italy), Korean (Korea), Lithuanian (Lithuania), Portuguese (Portugal), Spanish (USA), Thai (Thailand), Vietnamese (Vietnam)	https://eprovide.mapi-trust.org/instruments/pediatric-quality-of-life-inventory#languages
PeNAT	English	Dupuis (2006)
POQOLS	English	Goodwin (1994)
SSPedi	English, Spanish, French	https://www.sungresearch.com/sspedi
TRSC-C	English	Williams (2012)

Note. See Supplementary Table S4 for references for each PRO measure.

Supplementary Table S4. Key references for PRO measures included in the present review study for pediatric cancer populations.

PRO Measure	References
**Generic**	
BDI	Ambrosini, P.J.; Metz, C.; Bianchi, M.D.; Rabinovich, H.; Undie, A. Concurrent validity and psychometric properties of the Beck Depression Inventory in outpatient adolescents. *J Am Acad Child Adolesc Psychiatry* **1991**, *30*, 51-57, doi:10.1097/00004583-199101000-00008.<br>Beck, A.T. An inventory for measuring depression. *Arch Gen Psychiatry* **1961**, *4*, 561, doi:10.1001/archpsyc.1961.01710120031004.<br>Beck, A.T.; Steer, R.A.; Brown, G.K. *Manual for the Beck Depression Inventory-II*; Psychological Corporation: San Antonio, TX, 1996.<br>Beck, A.T.; Beck, R.W. Screening depressed patients in family practice. A rapid technic. *Postgrad Med* **1972**, *52*, 81-85, doi:10.1080/00325481.1972.11713319.<br>Beck, J.S; Beck, A.T.; Jolly, J.B; Steer, R.A. *Beck Youth Inventories for children and adolescents: Manual*; PsychCorp: San Antonio, TX, 2005.<br>Blackmon, J.E.; Liptak, C.; Recklitis, C.J. Development and preliminary validation of a short form of the Beck Depression Inventory for Youth (BDI-Y) in a sample of adolescent cancer survivors. *J Cancer Surviv* **2015**, *9*, 107-114, doi:10.1007/s11764-014-0394-y.<br>Blackmon, J.E.; Liptak, C.; Recklitis, C.J. Validation of three previously developed short forms of the Beck Depression Inventory for Youth: A replication. *J Adolesc Young Adult Oncol* **2017**, *6*, 190-192, doi:10.1089/jayao.2016.0052.<br>Tebbi, C.K.; Bromberg, C.; Mallon, J.C. Self-reported depression in adolescent cancer patients. *Am J Pediatr Hematol Oncol* **1988**, *10*, 185-190, doi:10.1097/00043426-198823000-00001.<br>Wu, P.C. Response shifts in depression intervention for early adolescents. *J Clin Psychol* **2016**, *72*, 663-675, doi:10.1002/jclp.22291.
CDI	Carey, M.P.; Faulstich, M.E.; Gresham, F.M.; Ruggiero, L.; Enyart, P. Children's Depression Inventory: Construct and discriminant validity across clinical and nonreferred (control) populations. *J Consult Clin Psychol* **1987**, *55*, 755-761, doi:10.1037//0022-006x.55.5.755.<br>Durualp, E.; Altay, N. A comparison of emotional indicators and depressive symptom levels of school-age children with and without cancer. *J Pediatr Oncol Nurs* **2012**, *29*, 232-239, doi:10.1177/1043454212446616.<br>Greenberg, H.S.; Kazak, A.E.; Meadows, A.T. Psychologic functioning in 8- to 16-year-old cancer survivors and their parents. *J Pediatr* **1989**, *114*, 488-493, doi:10.1016/s0022-3476(89)80581-5.<br>Kovacs, M. The Children's Depression, Inventory (CDI). *Psychopharmacol Bull* **1985**, *21*, 995-998.<br>Yoo, H.; Kim, D.S.; Shin, H.Y.; Lai, J.S.; Cella, D.; Park, H.J.; Ra, Y.S.; Kim, W.C.; Shin, Y.S. Validation of the Pediatric Functional Assessment of Cancer Therapy Questionnaire (Version 2.0) in brain tumor

	survivors aged 13 years and older. *J Pain Symptom Manage* **2010**, *40*, 559-565, doi:10.1016/j.jpainsymman.2010.01.024.
CHIP	Klosky, J.L.; Howell, C.R.; Li, Z.; Foster, R.H.; Mertens, A.C.; Robison, L.L.; Ness, K.K. Risky health behavior among adolescents in the Childhood Cancer Survivor Study cohort. *J Pediatr Psychol* **2012**, *37*, 634-646, doi:10.1093/jpepsy/jss046.<br>Riley, A.W.; Forrest, C.B.; Rebok, G.W.; Starfield, B.; Green, B.F.; Robertson, J.A.; Friello, P. The Child Report Form of the CHIP-Child Edition: Reliability and validity. *Med Care* **2004**, *42*, 221-231, doi:10.1097/01.mlr.0000114910.46921.73.<br>Riley, A.W.; Forrest, C.B.; Starfield, B.; Rebok, G.W.; Robertson, J.A.; Green, B.F. The Parent Report Form of the CHIP-Child Edition: Reliability and validity. *Med Care* **2004**, *42*, 210-220, doi:10.1097/01.mlr.0000114909.33878.ca.<br>Starfield, B.; Bergner, M.; Ensminger, M.; Riley, A.; Ryan, S.; Green, B.; McGauhey, P.; Skinner, A.; Kim, S. Adolescent health status measurement: Development of the Child Health and Illness Profile. *Pediatrics* **1993**, *91*, 430-435.<br>Starfield, B.; Riley, A.W.; Green, B.F.; Ensminger, M.E.; Ryan, S.A.; Kelleher, K.; Kim-Harris, S.; Johnston, D.; Vogel, K. The adolescent child health and illness profile. A population-based measure of health. *Med Care* **1995**, *33*, 553-566, doi:10.1097/00005650-199505000-00008.
CHQ	Banks, B.A.; Barrowman, N.J.; Klaassen, R. Health-related quality of life: Changes in children undergoing chemotherapy. *J Pediatr Hematol Oncol* **2008**, *30*, 292-297, doi:10.1097/MPH.0b013e3181647bda.<br>Bhatia, S.; Jenney, M.E.; Bogue, M.K.; Rockwood, T.H.; Feusner, J.H.; Friedman, D.L.; Robison, L.L.; Kane, R.L. The Minneapolis-Manchester Quality of Life instrument: Reliability and validity of the adolescent form. *J Clin Oncol* **2002**, *20*, 4692-4698, doi:10.1200/jco.2002.05.103.<br>Hepner, K.A.; Sechrest, L. Confirmatory factor analysis of the Child Health Questionnaire-Parent Form 50 in a predominantly minority sample. *Qual Life Res* **2002**, *11*, 763-773, doi:10.1023/a:1020822518857.<br>Landgraf, J.M.; Abetz, L.; Ware, J.E. *Child Health Questionnaire (CHQ): A user's manual*; Landgraf & Ware: 1999.<br>Landgraf, J.M.; van Grieken, A.; Raat, H. Giving voice to the child perspective: psychometrics and relative precision findings for the Child Health Questionnaire self-report short form (CHQ-CF45). *Qual Life Res* **2018**, *27*, 2165-2176, doi:10.1007/s11136-018-1873-9.<br>Nixon Speechley, K.; Maunsell, E.; Desmeules, M.; Schanzer, D.; Landgraf, J.M.; Feeny, D.H.; Barrera, M.E. Mutual concurrent validity of the Child Health Questionnaire and the Health Utilities Index: An exploratory analysis using survivors of childhood cancer. *Int J Cancer Suppl* **1999**, *12*, 95-105, doi:10.1002/(sici)1097-0215(1999)83:12+<95::aid-ijc18>3.0.co;2-h.<br>Sawyer, M.; Antoniou, G.; Toogood, I.; Rice, M. A comparison of parent and adolescent reports describing the health-related quality of life of adolescents treated for cancer. *Int J Cancer Suppl* **1999**, *12*, 39-45, doi:10.1002/(sici)1097-0215(1999)83:12+<39::aid-ijc8>3.0.co;2-7.

	Sung, L.; Greenberg, M.L.; Doyle, J.J.; Young, N.L.; Ingber, S.; Rubenstein, J.; Wong, J.; Samanta, T.; McLimont, M.; Feldman, B.M. Construct validation of the Health Utilities Index and the Child Health Questionnaire in children undergoing cancer chemotherapy. *Br J Cancer* **2003**, *88*, 1185-1190, doi:10.1038/sj.bjc.6600895.<br>Sung, L.; Greenberg, M.L.; Young, N.L.; McLimont, M.; Ingber, S.; Rubenstein, J.; Wong, J.; Samanta, T.; Doyle, J.J.; Stain, A.M.; et al. Validity of a modified standard gamble elicited from parents of a hospital-based cohort of children. *J Clin Epidemiol* **2003**, *56*, 848-855, doi:https://doi.org/10.1016/S0895-4356(03)00160-4.<br>Waters, E.B.; Wake, M.A.; Hesketh, K.D.; Ashley, D.M.; Smibert, E. Health-related quality of life of children with acute lymphoblastic leukaemia: Comparisons and correlations between parent and clinician reports. *Int J Cancer* **2003**, *103*, 514-518, doi:10.1002/ijc.10815.
DCGM	af Sandeberg, M.; Johansson, E.; Björk, O.; Wettergren, L. Health-related quality of life relates to school attendance in children on treatment for cancer. *J Pediatr Oncol Nurs* **2008**, *25*, 265-274, doi:10.1177/1043454208321119.<br>Baars, R.M.; Atherton, C.I.; Koopman, H.M.; Bullinger, M.; Power, M.; group, D. The European DISABKIDS project: Development of seven condition-specific modules to measure health related quality of life in children and adolescents. *Health Qual Life Outcomes* **2005**, *3*, 70-70, doi:10.1186/1477-7525-3-70.<br>Ravens-Sieberer, U.; Schmidt, S.; Gosch, A.; Erhart, M.; Petersen, C.; Bullinger, M. Measuring subjective health in children and adolescents: Results of the European KIDSCREEN/DISABKIDS Project. *Psychosoc Med* **2007**, *4*, Doc08-Doc08.<br>Santos, S.; Crespo, C.; Canavarro, M.C.; Fernandes, A.; Batalha, L.; de Campos, D.; Pinto, A. Psychometric study of the European Portuguese version of the PedsQL 3.0 Cancer Module. *Health Qual Life Outcomes* **2016**, *14*, 20, doi:10.1186/s12955-016-0421-y.<br>The DISABKIDS Group Europe. *The DISABKIDS questionnaires: Quality of life questionnaires for children with chronic conditions - Handbook*; Pabst Science Publishers: Lengerich, 2006.

FPSR	Baxter, A.L.; Watcha, M.F.; Baxter, W.V.; Leong, T.; Wyatt, M.M. Development and validation of a pictorial nausea rating scale for children. *Pediatrics* **2011**, *127*, e1542-1549, doi:10.1542/peds.2010-1410.<br>Bieri, D.; Reeve, R.A.; Champion, D.G.; Addicoat, L.; Ziegler, J.B. The Faces Pain Scale for the self-assessment of the severity of pain experienced by children: Development, initial validation, and preliminary investigation for ratio scale properties. *Pain* **1990**, *41*, 139-150, doi:10.1016/0304-3959(90)90018-9.<br>da Silva, F.C.; Thuler, L.C. Cross-cultural adaptation and translation of two pain assessment tools in children and adolescents. *J Pediatr (Rio J)* **2008**, *84*, 344-349, doi:10.2223/jped.1809.<br>Hicks, C.L.; von Baeyer, C.L.; Spafford, P.A.; van Korlaar, I.; Goodenough, B. The Faces Pain Scale-Revised: Toward a common metric in pediatric pain measurement. *Pain* **2001**, *93*, 173-183, doi:10.1016/s0304-3959(01)00314-1.<br>Spagrud, L.J.; von Baeyer, C.L.; Ali, K.; Mpofu, C.; Fennell, L.P.; Friesen, K.; Mitchell, J. Pain, distress, and adult-child interaction during venipuncture in pediatric oncology: An examination of three types of venous access. *J Pain Symptom Manage* **2008**, *36*, 173-184, doi:10.1016/j.jpainsymman.2007.10.009.
HADS	Annunziata, M.A.; Muzzatti, B.; Altoè, G. Defining hospital anxiety and depression scale (HADS) structure by confirmatory factor analysis: A contribution to validation for oncological settings. *Ann Oncol* **2011**, *22*, 2330-2333, doi:10.1093/annonc/mdq750.<br>Berard, R.M.; Boermeester, F. Psychiatric symptomatology in adolescents with cancer. *Pediatr Hematol Oncol* **1998**, *15*, 211-221, doi:10.3109/08880019809028787.<br>Jörngården, A.; Mattsson, E.; von Essen, L. Health-related quality of life, anxiety and depression among adolescents and young adults with cancer: A prospective longitudinal study. *Eur J Cancer* **2007**, *43*, 1952-1958, doi:10.1016/j.ejca.2007.05.031.<br>Moorey, S.; Greer, S.; Watson, M.; Gorman, C.; Rowden, L.; Tunmore, R.; Robertson, B.; Bliss, J. The factor structure and factor stability of the hospital anxiety and depression scale in patients with cancer. *Br J Psychiatry* **1991**, *158*, 255-259, doi:10.1192/bjp.158.2.255.<br>Zigmond, A.S.; Snaith, R.P. The Hospital Anxiety and Depression Scale. *Acta Psychiatr Scand* **1983**, *67*, 361-370, doi:https://doi.org/10.1111/j.1600-0447.1983.tb09716.x.
HUI	Banks, B.A.; Barrowman, N.J.; Klaassen, R. Health-related quality of life: Changes in children undergoing chemotherapy. *J Pediatr Hematol Oncol* **2008**, *30*, 292-297, doi:10.1097/MPH.0b013e3181647bda.<br>Barr, R.; Petrie, C.; Furlong, W.; Rothney, M.; Feeny, D. Health-related quality of life during post-induction chemotherapy in children with acute lymphoblastic leukemia in remission. *Int J Oncol* **1997**, *11*, 333-339, doi:10.3892/ijo.11.2.333.<br>Feeny, D.H., Torrance, George W., and Furlong, William J. . Health Utilities Index. In *Quality of Life and Pharmacoeconomics in Clinical Trials*, Second ed.; Spilker, B., Ed.; Lippincott-Raven Press: Philadelphia, 1996; pp. 239-252.

	Nixon Speechley, K.; Maunsell, E.; Desmeules, M.; Schanzer, D.; Landgraf, J.M.; Feeny, D.H.; Barrera, M.E. Mutual concurrent validity of the Child Health Questionnaire and the Health Utilities Index: An exploratory analysis using survivors of childhood cancer. *Int J Cancer Suppl* **1999**, *12*, 95-105, doi:10.1002/(sici)1097-0215(1999)83:12+<95::aid-ijc18>3.0.co;2-h.<br>Sung, L.; Greenberg, M.L.; Doyle, J.J.; Young, N.L.; Ingber, S.; Rubenstein, J.; Wong, J.; Samanta, T.; McLimont, M.; Feldman, B.M. Construct validation of the Health Utilities Index and the Child Health Questionnaire in children undergoing cancer chemotherapy. *Br J Cancer* **2003**, *88*, 1185-1190, doi:10.1038/sj.bjc.6600895.<br>Trudel, J.G.; Rivard, M.; Dobkin, P.L.; Leclerc, J.M.; Robaey, P. Psychometric properties of the Health Utilities Index Mark 2 system in paediatric oncology patients. *Qual Life Res* **1998**, *7*, 421-432, doi:10.1023/a:1008857920624.
KIDSCREEN	Engelen, V.; Koopman, H.M.; Detmar, S.B.; Raat, H.; van de Wetering, M.D.; Brons, P.; Anninga, J.K.; Abbink, F.; Grootenhuis, M.A. Health-related quality of life after completion of successful treatment for childhood cancer. *Pediatr Blood Cancer* **2011**, *56*, 646-653, doi:10.1002/pbc.22795.<br>Gillison, F.; Skevington, S.; Standage, M. Exploring response shift in the quality of life of healthy adolescents over 1 year. *Qual Life Res* **2008**, *17*, 997-1008, doi:10.1007/s11136-008-9373-y.<br>Jervaeus, A.; Lampic, C.; Johansson, E.; Malmros, J.; Wettergren, L. Clinical significance in self-rated HRQoL among survivors after childhood cancer – demonstrated by anchor-based thresholds. *Acta Oncol* **2014**, *53*, 486-492, doi:10.3109/0284186x.2013.844852.<br>Ravens-Sieberer, U.; Erhart, M.; Rajmil, L.; Herdman, M.; Auquier, P.; Bruil, J.; Power, M.; Duer, W.; Abel, T.; Czemy, L.; et al. Reliability, construct and criterion validity of the KIDSCREEN-10 score: A short measure for children and adolescents' well-being and health-related quality of life. *Qual Life Res* **2010**, *19*, 1487-1500, doi:10.1007/s11136-010-9706-5.<br>Ravens-Sieberer, U.; Gosch, A.; Rajmil, L.; Erhart, M.; Bruil, J.; Power, M.; Duer, W.; Auquier, P.; Cloetta, B.; Czemy, L.; et al. The KIDSCREEN-52 quality of life measure for children and adolescents: Psychometric results from a cross-cultural survey in 13 European countries. *Value Health* **2008**, *11*, 645-658, doi:10.1111/j.1524-4733.2007.00291.x.<br>The KIDSCREEN Group Europe. *The KIDSCREEN Questionnaires - Quality of life questionnaires for children and adolescents. Handbook*; Pabst Science Publishers: Lengerich, 2006.
KINDL	Benesch, M.; Spiegl, K.; Winter, A.; Passini, A.; Lackner, H.; Moser, A.; Sovinz, P.; Schwinger, W.; Urban, C. A scoring system to quantify late effects in children after treatment for medulloblastoma/ependymoma and its correlation with quality of life and neurocognitive functioning. *Childs Nerv Syst* **2009**, *25*, 173-181, doi:10.1007/s00381-008-0742-1.<br>Bullinger, M. KINDL. A questionnaire for health-related quality of life assessment in children. *Zetschrift fur Gesundheits psychologie* **1994**, *1*, 64-67.

	Ergin, D.; Eser, E.; Kantar, M.; Ekti Genç, R. Psychometric properties of the oncology module of the KINDL scale: First results. *J Pediatr Oncol Nurs* **2015**, *32*, 83-95, doi:10.1177/1043454214543020.<br>Müller, C.; Krauth, K.A.; Gerß, J.; Rosenbaum, D. Physical activity and health-related quality of life in pediatric cancer patients following a 4-week inpatient rehabilitation program. *Support Care Cancer* **2016**, *24*, 3793-3802, doi:10.1007/s00520-016-3198-y.<br>Ravens-Sieberer, U.; Bullinger, M. Assessing health-related quality of life in chronically ill children with the German KINDL: First psychometric and content analytical results. *Qual Life Res* **1998a**, *7*, 399-407, doi:10.1023/a:1008853819715.<br>Ravens-Sieberer, U.; Bullinger, M. News from the KINDL-questionnaire: A new version for adolescents. *Qual Life Res* **1998b**, 653-653.
PedsQL-Core	Banks, B.A.; Barrowman, N.J.; Klaassen, R. Health-related quality of life: Changes in children undergoing chemotherapy. *J Pediatr Hematol Oncol* **2008**, *30*, 292-297, doi:10.1097/MPH.0b013e3181647bda.<br>Brinksma, A.; Tissing, W.J.; Sulkers, E.; Kamps, W.A.; Roodbol, P.F.; Sanderman, R. Exploring the response shift phenomenon in childhood patients with cancer and its effect on health-related quality of life. *Oncol Nurs Forum* **2014**, *41*, 48-56, doi:10.1188/14.Onf.41-01ap.<br>Eiser, C.; Vance, Y.H.; Horne, B.; Glaser, A.; Galvin, H. The value of the PedsQLTM in assessing quality of life in survivors of childhood cancer. *Child Care Health Dev* **2003**, *29*, 95-102, doi:10.1046/j.1365-2214.2003.00318.x.<br>Hoffman, S.; Lambert, M.C.; Nelson, T.D.; Trout, A.L.; Epstein, M.H.; Pick, R. Confirmatory factor analysis of the PedsQL among youth in a residential treatment setting. *Qual Life Res* **2013**, *22*, 2151-2157, doi:10.1007/s11136-012-0327-z.<br>Huang, I.C.; Thompson, L.A.; Chi, Y.Y.; Knapp, C.A.; Revicki, D.A.; Seid, M.; Shenkman, E.A. The linkage between pediatric quality of life and health conditions: Establishing clinically meaningful cutoff scores for the PedsQL. *Value Health* **2009**, *12*, 773-781, doi:10.1111/j.1524-4733.2008.00487.x.<br>Lau, J.T.; Yu, X.N.; Chu, Y.; Shing, M.M.; Wong, E.M.; Leung, T.F.; Li, C.K.; Fok, T.F.; Mak, W.W. Validation of the Chinese version of the Pediatric Quality of Life Inventory (PedsQL) Cancer Module. *J Pediatr Psychol* **2010**, *35*, 99-109, doi:10.1093/jpepsy/jsp035.<br>Sato, I.; Higuchi, A.; Yanagisawa, T.; Mukasa, A.; Ida, K.; Sawamura, Y.; Sugiyama, K.; Saito, N.; Kumabe, T.; Terasaki, M.; et al. Development of the Japanese version of the Pediatric Quality of Life Inventory™ Brain Tumor Module. *Health Qual Life Outcomes* **2010**, *8*, 38, doi:10.1186/1477-7525-8-38.<br>Sato, I.; Higuchi, A.; Yanagisawa, T.; Mukasa, A.; Ida, K.; Sawamura, Y.; Sugiyama, K.; Saito, N.; Kumabe, T.; Terasaki, M.; et al. Factors influencing self- and parent-reporting health-related quality of life in children with brain tumors. *Qual Life Res* **2013**, *22*, 185-201, doi:10.1007/s11136-012-0137-3.

	Varni, J.W.; Burwinkle, T.M.; Katz, E.R.; Meeske, K.; Dickinson, P. The PedsQL in pediatric cancer: Reliability and validity of the Pediatric Quality of Life Inventory Generic Core Scales, Multidimensional Fatigue Scale, and Cancer Module. *Cancer* **2002**, *94*, 2090-2106, doi:10.1002/cncr.10428.<br>Varni, J.W.; Seid, M.; Rode, C.A. The PedsQL: Measurement model for the pediatric quality of life inventory. *Med Care* **1999**, *37*, 126-139, doi:10.1097/00005650-199902000-00003.<br>Veldhuijzen Van Zanten, S.E.M.; El-Khouly, F.E.; Jansen, M.H.A.; Bakker, D.P.; Sanchez Aliaga, E.; Haasbeek, C.J.A.; Wolf, N.I.; Zwaan, C.M.; Vandertop, W.P.; Van Vuurden, D.G.; et al. A phase I/II study of gemcitabine during radiotherapy in children with newly diagnosed diffuse intrinsic pontine glioma. *Journal of Neuro-Oncology* **2017**, *135*, 307-315, doi:10.1007/s11060-017-2575-9.
PedsQL-MFM	Nunes, M.D.R.; Jacob, E.; Bomfim, E.O.; Lopes-Junior, L.C.; de Lima, R.A.G.; Floria-Santos, M.; Nascimento, L.C. Fatigue and health related quality of life in children and adolescents with cancer. *Eur J Oncol Nurs* **2017**, *29*, 39-46, doi:10.1016/j.ejon.2017.05.001.<br>Palmer, S.N.; Meeske, K.A.; Katz, E.R.; Burwinkle, T.M.; Varni, J.W. The PedsQL Brain Tumor Module: Initial reliability and validity. *Pediatr Blood Cancer* **2007**, *49*, 287-293, doi:10.1002/pbc.21026.<br>Tomlinson, D.; Hendershot, E.; Bartels, U.; Maloney, A.M.; Armstrong, C.; Wrathall, G.; Sung, L. Concordance between couples reporting their child's quality of life and their decision making in pediatric oncology palliative care. *J Pediatr Oncol Nurs* **2011**, *28*, 319-325, doi:10.1177/1043454211418666.<br>Varni, J.W.; Burwinkle, T.M.; Katz, E.R.; Meeske, K.; Dickinson, P. The PedsQL in pediatric cancer: Reliability and validity of the Pediatric Quality of Life Inventory Generic Core Scales, Multidimensional Fatigue Scale, and Cancer Module. *Cancer* **2002**, *94*, 2090-2106, doi:10.1002/cncr.10428.<br>Veldhuijzen Van Zanten, S.E.M.; El-Khouly, F.E.; Jansen, M.H.A.; Bakker, D.P.; Sanchez Aliaga, E.; Haasbeek, C.J.A.; Wolf, N.I.; Zwaan, C.M.; Vandertop, W.P.; Van Vuurden, D.G.; et al. A phase I/II study of gemcitabine during radiotherapy in children with newly diagnosed diffuse intrinsic pontine glioma. *Journal of Neuro-Oncology* **2017**, *135*, 307-315, doi:10.1007/s11060-017-2575-9.
PPS	Batra, P.; Kumar, B.; Gomber, S.; Bhatia, M.S. Assessment of quality of life during treatment of pediatric oncology patients. *Indian J Public Health* **2014**, *58*, 168-173, doi:10.4103/0019-557x.138623.<br>Eiser, C.; Havermans, T.; Craft, A.; Kernahan, J. Validity of the Rotterdam Symptom Checklist in paediatric oncology. *Med Pediatr Oncol* **1997**, *28*, 451-454, doi:10.1002/(sici)1096-911x(199706)28:6<451::aid-mpo11>3.0.co;2-c.<br>Klaassen, R.J.; Krahn, M.; Gaboury, I.; Hughes, J.; Anderson, R.; Grundy, P.; Ali, S.K.; Jardine, L.; Abla, O.; Silva, M.; et al. Evaluating the ability to detect change of health-related quality of life in children with Hodgkin disease. *Cancer* **2010**, *116*, 1608-1614, doi:10.1002/cncr.24883.<br>Lansky, L.L.; List, M.A.; Lansky, S.B.; Cohen, M.E.; Sinks, L.F. Toward the development of a play performance scale for children (PPSC). *Cancer* **1985**, *56*, 1837-1840, doi:10.1002/1097-0142(19851001)56:7+<1837::aid-cncr2820561324>3.0.co;2-z.

	Lansky, S.B.; List, M.A.; Lansky, L.L.; Ritter-Sterr, C.; Miller, D.R. The measurement of performance in childhood cancer patients. *Cancer* **1987**, *60*, 1651-1656, doi:https://doi.org/10.1002/1097-0142(19871001)60:7<1651::AID-CNCR2820600738>3.0.CO;2-J.
PROMIS	Hinds, P.S.; Nuss, S.L.; Ruccione, K.S.; Withycombe, J.S.; Jacobs, S.; DeLuca, H.; Faulkner, C.; Liu, Y.; Cheng, Y.I.; Gross, H.E.; et al. PROMIS pediatric measures in pediatric oncology: Valid and clinically feasible indicators of patient-reported outcomes. *Pediatr Blood Cancer* **2013**, *60*, 402-408, doi:10.1002/pbc.24233.<br>Hinds, P.S.; Wang, J.; Cheng, Y.I.; Stern, E.; Waldron, M.; Gross, H.; DeWalt, D.A.; Jacobs, S.S. PROMIS pediatric measures validated in a longitudinal study design in pediatric oncology. *Pediatr Blood Cancer* **2019**, *66*, e27606, doi:10.1002/pbc.27606.<br>Irwin, D.E.; Varni, J.W.; Yeatts, K.; DeWalt, D.A. Cognitive interviewing methodology in the development of a pediatric item bank: A Patient Reported Outcomes Measurement Information System (PROMIS) study. *Health Qual Life Outcomes* **2009**, *7*, 3, doi:10.1186/1477-7525-7-3.<br>Liu, Y.; Wang, J.; Hinds, P.S.; Wang, J.; Shen, N.; Zhao, X.; Ding, J.; Yuan, C. The emotional distress of children with cancer in China: An item response analysis of C-Ped-PROMIS Anxiety and Depression short forms. *Qual Life Res* **2015**, *24*, 1491-1501, doi:10.1007/s11136-014-0870-x.<br>Liu, Y.; Yuan, C.; Wang, J.; Shen, N.; Shen, M.; Hinds, P.S. Chinese version of Pediatric Patient-Reported Outcomes Measurement Information System short form measures: Reliability, validity, and factorial structure assessment in children With cancer in China. *Cancer Nurs* **2019**, *42*, 430-438, doi:10.1097/ncc.0000000000000633.<br>Reeve, B.B.; Edwards, L.J.; Jaeger, B.C.; Hinds, P.S.; Dampier, C.; Gipson, D.S.; Selewski, D.T.; Troost, J.P.; Thissen, D.; Barry, V.; et al. Assessing responsiveness over time of the PROMIS® pediatric symptom and function measures in cancer, nephrotic syndrome, and sickle cell disease. *Qual Life Res* **2018**, *27*, 249-257, doi:10.1007/s11136-017-1697-z.<br>Thissen, D.; Liu, Y.; Magnus, B.; Quinn, H.; Gipson, D.S.; Dampier, C.; Huang, I.C.; Hinds, P.S.; Selewski, D.T.; Reeve, B.B.; et al. Estimating minimally important difference (MID) in PROMIS pediatric measures using the scale-judgment method. *Qual Life Res* **2016**, *25*, 13-23, doi:10.1007/s11136-015-1058-8.<br>Walsh, T.R.; Irwin, D.E.; Meier, A.; Varni, J.W.; DeWalt, D.A. The use of focus groups in the development of the PROMIS pediatrics item bank. *Qual Life Res* **2008**, *17*, 725-735, doi:10.1007/s11136-008-9338-1.<br>Westmoreland, K.; Reeve, B.B.; Amuquandoh, A.; van der Gronde, T.; Manthalu, O.; Correia, H.; Stanley, C.; Itimu, S.; Salima, A.; Chikasema, M.; et al. Translation, psychometric validation, and baseline results of the Patient-Reported Outcomes Measurement Information System (PROMIS) pediatric measures to assess health-related quality of life of patients with pediatric lymphoma in Malawi. *Pediatr Blood Cancer* **2018**, *65*, e27353, doi:10.1002/pbc.27353.

RCMAS	Castaneda, A.; McCandless, B.R.; Palermo, D.S. The children's form of the Manifest Anxiety Scale. *Child Dev* **1956**, *27*.<br>Reynolds, C.R.; Richmond, B.O. What I think and feel: A revised measure of children's manifest anxiety. *J Abnorm Child Psychol* **1978**, *6*, 271-280, doi:10.1007/bf00919131.<br>Reynolds, C.R., Richmond, B. O. . *Revised Children's Manifest Anxiety Scale–Second Edition (RCMAS-2)*; Western Psychological Services: Los Angeles, CA, 2008.<br>Santos, S.; Crespo, C.; Canavarro, M.C.; Fernandes, A.; Batalha, L.; de Campos, D.; Pinto, A. Psychometric study of the European Portuguese version of the PedsQL 3.0 Cancer Module. *Health Qual Life Outcomes* **2016**, *14*, 20, doi:10.1186/s12955-016-0421-y.<br>von Essen, L.; Enskär, K.; Kreuger, A.; Larsson, B.; Sjödén, P.O. Self-esteem, depression and anxiety among Swedish children and adolescents on and off cancer treatment. *Acta Paediatr* **2000**, *89*, 229-236, doi:10.1080/080352500750028889.<br>Wu, P.C. Response shifts in depression intervention for early adolescents. *J Clin Psychol* **2016**, *72*, 663-675, doi:10.1002/jclp.22291.
STAIC	Allen, R.; Newman, S.P.; Souhami, R.L. Anxiety and depression in adolescent cancer: Findings in patients and parents at the time of diagnosis. *Eur J Cancer* **1997**, *33*, 1250-1255, doi:10.1016/s0959-8049(97)00176-7.<br>Maurice-Stam, H.; Broek, A.; Kolk, A.M.; Vrijmoet-Wiersma, J.M.; Meijer-van den Bergh, E.; van Dijk, E.M.; Phipps, S.; Grootenhuis, M.A. Measuring perceived benefit and disease-related burden in young cancer survivors: Validation of the Benefit and Burden Scale for Children (BBSC) in The Netherlands. *Support Care Cancer* **2011**, *19*, 1249-1253, doi:10.1007/s00520-011-1206-9.<br>Moore, J.B.; Mosher, R.B. Adjustment responses of children and their mothers to cancer: Self-care and anxiety. *Oncol Nurs Forum* **1997**, *24*, 519-525.<br>Sato, I.; Higuchi, A.; Yanagisawa, T.; Mukasa, A.; Ida, K.; Sawamura, Y.; Sugiyama, K.; Saito, N.; Kumabe, T.; Terasaki, M.; et al. Development of the Japanese version of the Pediatric Quality of Life Inventory™ Brain Tumor Module. *Health Qual Life Outcomes* **2010**, *8*, 38, doi:10.1186/1477-7525-8-38.<br>Sato, I.; Higuchi, A.; Yanagisawa, T.; Mukasa, A.; Ida, K.; Sawamura, Y.; Sugiyama, K.; Saito, N.; Kumabe, T.; Terasaki, M.; et al. Factors influencing self- and parent-reporting health-related quality of life in children with brain tumors. *Qual Life Res* **2013**, *22*, 185-201, doi:10.1007/s11136-012-0137-3.<br>Spielberger, C.D. *The state-trait anxiety inventory for children (STAIC)*; The Psychological Corporation: San Antonio, 1973.
TNO-AZL	Fekkes, M.; Theunissen, N.C.; Brugman, E.; Veen, S.; Verrips, E.G.; Koopman, H.M.; Vogels, T.; Wit, J.M.; Verloove-Vanhorick, S.P. Development and psychometric evaluation of the TAPQOL: A health-related quality of life instrument for 1-5-year-old children. *Qual Life Res* **2000**, *9*, 961-972, doi:10.1023/a:1008981603178.

	Koopman, H.M.; Koetsier, J.A.; Taminiau, A.H.; Hijnen, K.E.; Bresters, D.; Egeler, R.M. Health-related quality of life and coping strategies of children after treatment of a malignant bone tumor: A 5-year follow-up study. *Pediatr Blood Cancer* **2005**, *45*, 694-699, doi:10.1002/pbc.20408.<br>Landolt, M.A.; Vollrath, M.; Niggli, F.K.; Gnehm, H.E.; Sennhauser, F.H. Health-related quality of life in children with newly diagnosed cancer: A one year follow-up study. *Health Qual Life Outcomes* **2006**, *4*, 63, doi:10.1186/1477-7525-4-63.<br>Maurice-Stam, H.; Oort, F.J.; Last, B.F.; Brons, P.P.; Caron, H.N.; Grootenhuis, M.A. Longitudinal assessment of health-related quality of life in preschool children with non-CNS cancer after the end of successful treatment. *Pediatr Blood Cancer* **2008**, *50*, 1047-1051, doi:10.1002/pbc.21374.<br>Verrips, E.G.H.; Vogels, T.G.C.; Koopman, H.M.; Theunissen, N.C.M.; Kamphuis, R.P.; Fekkes, M.; Wit, J.M.; Vanhorick, S.P.V. Measuring health-related quality of life in a child population. *Eur J Public Health* **1999**, *9*, 188-193, doi:10.1093/eurpub/9.3.188.<br>Vogels, T.; Verrips, G.H.; Verloove-Vanhorick, S.P.; Fekkes, M.; Kamphuis, R.P.; Koopman, H.M.; Theunissen, N.C.; Wit, J.M. Measuring health-related quality of life in children: The development of the TACQOL parent form. *Qual Life Res* **1998**, *7*, 457-465, doi:10.1023/a:1008848218806.
**Cancer-specific**	
BASES	Mehling, W.E.; Lown, E.A.; Dvorak, C.C.; Cowan, M.J.; Horn, B.N.; Dunn, E.A.; Acree, M.; Abrams, D.I.; Hecht, F.M. Hematopoietic Cell Transplant and Use of Massage for Improved Symptom Management: Results from a Pilot Randomized Control Trial. *Evidence-Based Complementary and Alternative Medicine* **2012**, *2012*, 1-9, doi:10.1155/2012/450150.<br>Phipps, S.; Dunavant, M.; Jayawardene, D.; Srivastiva, D.K. Assessment of health-related quality of life in acute in-patient settings: Use of the BASES instrument in children undergoing bone marrow transplantation. *Int J Cancer Suppl* **1999**, *12*, 18-24, doi:10.1002/(sici)1097-0215(1999)83:12+<18::aid-ijc5>3.0.co;2-l.<br>Phipps, S.; Hinds, P.S.; Channell, S.; Bell, G.L. Measurement of behavioral, affective, and somatic responses to pediatric bone marrow transplantation: Development of the BASES scale. *J Pediatr Oncol Nurs* **1994**, *11*, 109-117; discussion 118-109, doi:10.1177/104345429401100305.<br>Rosipal, N.C.; Mingle, L.; Smith, J.; Morris, G.S. Assessment of Voluntary Exercise Behavior and Active Video Gaming Among Adolescent and Young Adult Patients During Hematopoietic Stem Cell Transplantation. *Journal of Pediatric Oncology Nursing* **2013**, *30*, 24-33, doi:10.1177/1043454212461071.<br>Ullrich, C.K.; Rodday, A.M.; Bingen, K.M.; Kupst, M.J.; Patel, S.K.; Syrjala, K.L.; Harris, L.L.; Recklitis, C.J.; Chang, G.; Guinan, E.C.; et al. Three sides to a story: Child, parent, and nurse perspectives on the child's experience during hematopoietic stem cell transplantation. *Cancer* **2017**, *123*, 3159-3166, doi:10.1002/cncr.30723.

ChIMES	Jacobs, S.; Baggott, C.; Agarwal, R.; Hesser, T.; Schechter, T.; Judd, P.; Tomlinson, D.; Beyene, J.; Sung, L. Validation of the Children's International Mucositis Evaluation Scale (ChIMES) in paediatric cancer and SCT. *Br J Cancer* **2013**, *109*, 2515-2522, doi:10.1038/bjc.2013.618.<br>Khurana, H.; Pandey, R.K.; Saksena, A.K.; Kumar, A. An evaluation of Vitamin E and Pycnogenol in children suffering from oral mucositis during cancer chemotherapy. *Oral Dis* **2013**, *19*, 456-464, doi:10.1111/odi.12024.<br>Paiva, B.S.R.; Barroso, E.M.; Cadamuro, S.A.; Paula, L.A.B.; Pirola, W.E.; Serrano, C.; Paiva, C.E. The Children's International Mucositis Evaluation Scale is valid and reliable for the assessment of mucositis among Brazilian children with cancer. *J Pain Symptom Manage* **2018**, *56*, 774-780.e772, doi:10.1016/j.jpainsymman.2018.07.015.<br>Tomlinson, D.; Gibson, F.; Treister, N.; Baggott, C.; Judd, P.; Hendershot, E.; Maloney, A.M.; Doyle, J.; Feldman, B.; Sung, L. Designing an oral mucositis assessment instrument for use in children: Generating items using a nominal group technique. *Support Care Cancer* **2009**, *17*, 555-562, doi:10.1007/s00520-008-0523-0.<br>Tomlinson, D.; Gibson, F.; Treister, N.; Baggott, C.; Judd, P.; Hendershot, E.; Maloney, A.-M.; Doyle, J.; Feldman, B.; Kwong, K.; et al. Understandability, content validity, and overall acceptability of the Children's International Mucositis Evaluation Scale (ChIMES): Child and parent reporting. *J Pediatr Hematol Oncol* **2009**, *31*, 416-423, doi:10.1097/MPH.0b013e31819c21ab.
FS	Chiang, Y.C.; Hinds, P.S.; Yeh, C.H.; Yang, C.P.; Srivastava, D.K. Reliability and validity of the Chinese version of the Fatigue Scale-Adolescent. *Cancer Nurs* **2008**, *31*, E1-8, doi:10.1097/01.Ncc.0000305732.03464.29.<br>Hinds, P.S.; Hockenberry-Eaton, M.; Gilger, E.; Kline, N.; Burleson, C.; Bottomley, S.; Quargnenti, A. Comparing patient, parent, and staff descriptions of fatigue in pediatric oncology patients. *Cancer Nurs* **1999**, *22*, 277-288; quiz 288-279, doi:10.1097/00002820-199908000-00004.<br>Hinds, P.S.; Hockenberry, M.; Tong, X.; Rai, S.N.; Gattuso, J.S.; McCarthy, K.; Pui, C.H.; Srivastava, D.K. Validity and reliability of a new instrument to measure cancer-related fatigue in adolescents. *J Pain Symptom Manage* **2007**, *34*, 607-618, doi:10.1016/j.jpainsymman.2007.01.009.<br>Hinds, P.S.; Yang, J.; Gattuso, J.S.; Hockenberry, M.; Jones, H.; Zupanec, S.; Li, C.; Crabtree, V.M.; Mandrell, B.N.; Schoumacher, R.A.; et al. Psychometric and clinical assessment of the 10-item reduced version of the Fatigue Scale-Child instrument. *J Pain Symptom Manage* **2010**, *39*, 572-578, doi:10.1016/j.jpainsymman.2009.07.015.<br>Hockenberry, M.J.; Hinds, P.S.; Barrera, P.; Bryant, R.; Adams-McNeill, J.; Hooke, C.; Rasco-Baggott, C.; Patterson-Kelly, K.; Gattuso, J.S.; Manteuffel, B. Three instruments to assess fatigue in children with cancer: The child, parent and staff perspectives. *J Pain Symptom Manage* **2003**, *25*, 319-328, doi:10.1016/s0885-3924(02)00680-2.

	Mandrell, B.N.; Yang, J.; Hooke, M.C.; Wang, C.; Gattuso, J.S.; Hockenberry, M.; Jones, H.; Zupanec, S.; Hinds, P.S. Psychometric and clinical assessment of the 13-item reduced version of the Fatigue Scale-Adolescent instrument. *J Pediatr Oncol Nurs* **2011**, *28*, 287-294, doi:10.1177/1043454211418667.
MMQL	Bhatia, S.; Jenney, M.E.; Bogue, M.K.; Rockwood, T.H.; Feusner, J.H.; Friedman, D.L.; Robison, L.L.; Kane, R.L. The Minneapolis-Manchester Quality of Life instrument: Reliability and validity of the adolescent form. *J Clin Oncol* **2002**, *20*, 4692-4698, doi:10.1200/jco.2002.05.103.<br>Bhatia, S.; Jenney, M.E.; Wu, E.; Bogue, M.K.; Rockwood, T.H.; Feusner, J.H.; Friedman, D.L.; Robison, L.L.; Kane, R.L. The Minneapolis-Manchester Quality of Life instrument: Reliability and validity of the youth form. *J Pediatr* **2004**, *145*, 39-46, doi:10.1016/j.jpeds.2004.02.034.<br>Einberg, E.L.; Kadrija, I.; Brunt, D.; Nygren, J.N.; Svedberg, P. Psychometric evaluation of a Swedish version of Minneapolis-Manchester quality of life-youth form and adolescent form. *Health Qual Life Outcomes* **2013**, *11*, 79, doi:10.1186/1477-7525-11-79.<br>Hutchings, H.A.; Upton, P.; Cheung, W.Y.; Maddocks, A.; Eiser, C.; Williams, J.G.; Russell, I.T.; Jackson, S.; Jenney, M.E. Adaptation of the Manchester-Minneapolis Quality of Life instrument for use in the UK population. *Arch Dis Child* **2007**, *92*, 855-860, doi:10.1136/adc.2006.098947.<br>Koike, M.; Hori, H.; Rikiishi, T.; Hayakawa, A.; Tsuji, N.; Yonemoto, T.; Uryu, H.; Matsushima, E. Development of the Japanese version of the Minneapolis-Manchester Quality of Life Survey of Health - Adolescent Form (MMQL-AF) and investigation of its reliability and validity. *Health Qual Life Outcomes* **2014**, *12*, 127, doi:10.1186/s12955-014-0127-y.<br>Shankar, S.; Robison, L.; Jenney, M.E.; Rockwood, T.H.; Wu, E.; Feusner, J.; Friedman, D.; Kane, R.L.; Bhatia, S. Health-related quality of life in young survivors of childhood cancer using the Minneapolis-Manchester Quality of Life-Youth Form. *Pediatrics* **2005**, *115*, 435-442, doi:10.1542/peds.2004-0649.
MSAS	Chang, V.T.; Hwang, S.S.; Feuerman, M.; Kasimis, B.S.; Thaler, H.T. The memorial symptom assessment scale short form (MSAS-SF). *Cancer* **2000**, *89*, 1162-1171, doi:10.1002/1097-0142(20000901)89:5<1162::aid-cncr26>3.0.co;2-y.<br>Collins, J.J.; Byrnes, M.E.; Dunkel, I.J.; Lapin, J.; Nadel, T.; Thaler, H.T.; Polyak, T.; Rapkin, B.; Portenoy, R.K. The measurement of symptoms in children with cancer. *J Pain Symptom Manage* **2000**, *19*, 363-377, doi:10.1016/s0885-3924(00)00127-5.<br>Collins, J.J.; Devine, T.D.; Dick, G.S.; Johnson, E.A.; Kilham, H.A.; Pinkerton, C.R.; Stevens, M.M.; Thaler, H.T.; Portenoy, R.K. The measurement of symptoms in young children with cancer: The validation of the Memorial Symptom Assessment Scale in children aged 7-12. *J Pain Symptom Manage* **2002**, *23*, 10-16, doi:10.1016/s0885-3924(01)00375-x.<br>Li, R.; Ma, J.; Chan, Y.; Yang, Q.; Zhang, C. Symptom clusters and influencing factors in children with acute leukemia during chemotherapy. *Cancer Nurs* **2020**, *43*, 411-418, doi:10.1097/ncc.0000000000000716.

	Portenoy, R.K.; Thaler, H.T.; Kornblith, A.B.; Lepore, J.M.; Friedlander-Klar, H.; Kiyasu, E.; Sobel, K.; Coyle, N.; Kemeny, N.; Norton, L.; et al. The Memorial Symptom Assessment Scale: An instrument for the evaluation of symptom prevalence, characteristics and distress. *Eur J Cancer* **1994**, *30a*, 1326-1336, doi:10.1016/0959-8049(94)90182-1.
OMDQ	Cheng, K.K.F.; Ip, W.Y.; Lee, V.; Li, C.H.; Yuen, H.L.; Epstein, J.B. Measuring Oral Mucositis of Pediatric Patients with Cancer: A Psychometric Evaluation of Chinese Version of the Oral Mucositis Daily Questionnaire. *Asia Pac J Oncol Nurs* **2017**, *4*, 330-335, doi:10.4103/apjon.apjon_39_17.<br>Jacobs, S.; Baggott, C.; Agarwal, R.; Hesser, T.; Schechter, T.; Judd, P.; Tomlinson, D.; Beyene, J.; Sung, L. Validation of the Children's International Mucositis Evaluation Scale (ChIMES) in paediatric cancer and SCT. *Br J Cancer* **2013**, *109*, 2515-2522, doi:10.1038/bjc.2013.618.<br>Paiva, B.S.R.; Barroso, E.M.; Cadamuro, S.A.; Paula, L.A.B.; Pirola, W.E.; Serrano, C.; Paiva, C.E. The Children's International Mucositis Evaluation Scale is valid and reliable for the assessment of mucositis among Brazilian children with cancer. *J Pain Symptom Manage* **2018**, *56*, 774-780.e772, doi:10.1016/j.jpainsymman.2018.07.015.<br>Stiff, P.J.; Erder, H.; Bensinger, W.I.; Emmanouilides, C.; Gentile, T.; Isitt, J.; Lu, Z.J.; Spielberger, R. Reliability and validity of a patient self-administered daily questionnaire to assess impact of oral mucositis (OM) on pain and daily functioning in patients undergoing autologous hematopoietic stem cell transplantation (HSCT). *Bone Marrow Transplant* **2006**, *37*, 393-401, doi:10.1038/sj.bmt.1705250.<br>Tomlinson, D.; Ethier, M.C.; Judd, P.; Doyle, J.; Gassas, A.; Naqvi, A.; Sung, L. Reliability and construct validity of the Oral Mucositis Daily Questionnaire in children with cancer. *Eur J Cancer* **2011**, *47*, 383-388, doi:10.1016/j.ejca.2010.09.018.
Pain Squad App	Jibb, L.A.; Cafazzo, J.A.; Nathan, P.C.; Seto, E.; Stevens, B.J.; Nguyen, C.; Stinson, J.N. Development of a mHealth real-time pain self-management app for adolescents with cancer: An iterative usability testing study [Formula: see text]. *J Pediatr Oncol Nurs* **2017**, *34*, 283-294, doi:10.1177/1043454217697022.<br>Jibb, L.A.; Stevens, B.J.; Nathan, P.C.; Seto, E.; Cafazzo, J.A.; Johnston, D.L.; Hum, V.; Stinson, J.N. Implementation and preliminary effectiveness of a real-time pain management smartphone app for adolescents with cancer: A multicenter pilot clinical study. *Pediatr Blood Cancer* **2017**, *64*, doi:10.1002/pbc.26554.<br>Jibb, L.A.; Stevens, B.J.; Nathan, P.C.; Seto, E.; Cafazzo, J.A.; Johnston, D.L.; Hum, V.; Stinson, J.N. Perceptions of Adolescents With Cancer Related to a Pain Management App and Its Evaluation: Qualitative Study Nested Within a Multicenter Pilot Feasibility Study. *JMIR Mhealth Uhealth* **2018**, *6*, e80, doi:10.2196/mhealth.9319.<br>Stinson, J.N.; Jibb, L.A.; Nguyen, C.; Nathan, P.C.; Maloney, A.M.; Dupuis, L.L.; Gerstle, J.T.; Alman, B.; Hopyan, S.; Strahlendorf, C.; et al. Development and testing of a multidimensional iPhone pain

	assessment application for adolescents with cancer. *J Med Internet Res* **2013**, *15*, e51, doi:10.2196/jmir.2350.<br>Stinson, J.N.; Jibb, L.A.; Nguyen, C.; Nathan, P.C.; Maloney, A.M.; Dupuis, L.L.; Gerstle, J.T.; Hopyan, S.; Alman, B.A.; Strahlendorf, C.; et al. Construct validity and reliability of a real-time multidimensional smartphone app to assess pain in children and adolescents with cancer. *Pain* **2015**, *156*, 2607-2615, doi:10.1097/j.pain.0000000000000385.<br>Tutelman, P.R.; Chambers, C.T.; Stinson, J.N.; Parker, J.A.; Barwick, M.; Witteman, H.O.; Jibb, L.; Stinson, H.C.; Fernandez, C.V.; Nathan, P.C.; et al. The Implementation Effectiveness of a Freely Available Pediatric Cancer Pain Assessment App: A Pilot Implementation Study. *JMIR Cancer* **2018**, *4*, e10280, doi:10.2196/10280.
PCQL-32	Seid, M.; Varni, J.W.; Rode, C.A.; Katz, E.R. The Pediatric Cancer Quality of Life Inventory: A modular approach to measuring health-related quality of life in children with cancer. *Int J Cancer Suppl* **1999**, *12*, 71-76, doi:10.1002/(sici)1097-0215(1999)83:12+<71::aid-ijc13>3.0.co;2-5.<br>Varni, J.W.; Katz, E.R.; Seid, M.; Quiggins, D.J.; Friedman-Bender, A. The pediatric cancer quality of life inventory-32 (PCQL-32): I. Reliability and validity. *Cancer* **1998a**, *82*, 1184-1196, doi:10.1002/(sici)1097-0142(19980315)82:6<1184::aid-cncr25>3.0.co;2-1.<br>Varni, J.W.; Katz, E.R.; Seid, M.; Quiggins, D.J.; Friedman-Bender, A.; Castro, C.M. The Pediatric Cancer Quality of Life Inventory (PCQL). I. Instrument development, descriptive statistics, and cross-informant variance. *J Behav Med* **1998b**, *21*, 179-204, doi:10.1023/a:1018779908502.<br>Varni, J.W.; Rode, C.A.; Seid, M.; Katz, E.R.; Friedman-Bender, A.; Quiggins, D.J.L. The Pediatric Cancer Quality of Life Inventory-32 (PCQL-32). II. Feasibility and range of measurement. *Journal of Behavioral Medicine* **1999**, *22*, 397-406, doi:10.1023/a:1018730204210.<br>Waters, E.B.; Wake, M.A.; Hesketh, K.D.; Ashley, D.M.; Smibert, E. Health-related quality of life of children with acute lymphoblastic leukaemia: Comparisons and correlations between parent and clinician reports. *Int J Cancer* **2003**, *103*, 514-518, doi:10.1002/ijc.10815.
PEDQOL	Calaminus, G.; Weinspach, S.; Teske, C.; Göbel, U. Quality of life in children and adolescents with cancer. First results of an evaluation of 49 patients with the PEDQOL questionnaire. *Klin Padiatr* **2000**, *212*, 211-215, doi:10.1055/s-2000-9679.<br>Calaminus, G.; Weinspach, S.; Teske, C.; Göbel, U. Quality of survival in children and adolescents after treatment for childhood cancer: The influence of reported late effects on health related quality of life. *Klin Padiatr* **2007**, *219*, 152-157, doi:10.1055/s-2007-973846.<br>Heinks, K.; Boekhoff, S.; Hoffmann, A.; Warmuth-Metz, M.; Eveslage, M.; Peng, J.; Calaminus, G.; Müller, H.L. Quality of life and growth after childhood craniopharyngioma: Results of the multinational trial KRANIOPHARYNGEOM 2007. *Endocrine* **2018**, *59*, 364-372, doi:10.1007/s12020-017-1489-9.<br>Müller, H.L.; Bueb, K.; Bartels, U.; Roth, C.; Harz, K.; Graf, N.; Korinthenberg, R.; Bettendorf, M.; Kühl, J.; Gutjahr, P.; et al. Obesity after childhood craniopharyngioma - German multicenter study on pre-

	operative risk factors and quality of life. *Klinische Pädiatrie* **2001**, *213*, 244-249, doi:10.1055/s-2001-16855.<br>Seifert, G.; Calaminus, G.; Wiener, A.; Cysarz, D. Heart rate variability reflects the natural history of physiological development in healthy children and is not associated with quality of life. *PLoS ONE* **2014**, *9*, e91036, doi:10.1371/journal.pone.0091036.
PedsFACT-Brs	Lai, J.S.; Cella, D.; Tomita, T.; Bode, R.K.; Newmark, M.; Goldman, S. Developing a health-related quality of life instrument for childhood brain tumor survivors. *Childs Nerv Syst* **2007**, *23*, 47-57, doi:10.1007/s00381-006-0176-6.<br>Yoo, H.-J.; Kim, D.-S.; Lai, J.-S.; Cella, D.; Shin, H.-Y.; Ra, Y.-S. Validation of Pediatric Functional Assessment of Cancer Therapy Questionnaire (Version 2.0) in Brain Tumor Survivor Aged 13 Years and Older (Parent Form)(PedsFACT-BrS Parent of Adolescent). *Journal of Korean Neurosurgical Society* **2011**, *49*, 147, doi:10.3340/jkns.2011.49.3.147.<br>Yoo, H.; Kim, D.S.; Shin, H.Y.; Lai, J.S.; Cella, D.; Park, H.J.; Ra, Y.S.; Kim, W.C.; Shin, Y.S. Validation of the Pediatric Functional Assessment of Cancer Therapy Questionnaire (Version 2.0) in brain tumor survivors aged 13 years and older. *J Pain Symptom Manage* **2010**, *40*, 559-565, doi:10.1016/j.jpainsymman.2010.01.024.<br>Yoo, H.-J.; Ra, Y.-S.; Park, H.-J.; Lai, J.-S.; Cella, D.; Shin, H.-Y.; Kim, D.-S. Agreement between pediatric brain tumor patients and parent proxy reports regarding the Pediatric Functional Assessment of Cancer Therapy-Childhood Brain Tumor Survivors questionnaire, version 2. *Cancer* **2010**, *116*, 3674-3682, doi:10.1002/cncr.25200.<br>Yoo, H.; Ra, Y.S.; Park, H.J.; Lai, J.S.; Cella, D.; Shin, H.Y.; Kim, D.S.; Kim, W.C.; Shin, Y.S. Validation of pediatric Functional Assessment of Cancer Therapy: Patient version 2 of "brain tumor survivor" for grade school patients aged 7-12 years. *Qual Life Res* **2011**, *20*, 529-535, doi:10.1007/s11136-010-9786-2.
PedsQL-Brain Tumor	Kuhlthau, K.A.; Pulsifer, M.B.; Yeap, B.Y.; Rivera Morales, D.; Delahaye, J.; Hill, K.S.; Ebb, D.; Abrams, A.N.; Macdonald, S.M.; Tarbell, N.J.; et al. Prospective Study of Health-Related Quality of Life for Children With Brain Tumors Treated With Proton Radiotherapy. *Journal of Clinical Oncology* **2012**, *30*, 2079-2086, doi:10.1200/jco.2011.37.0577.<br>Mandrell, B.N.; Baker, J.; Levine, D.; Gattuso, J.; West, N.; Sykes, A.; Gajjar, A.; Broniscer, A. Children with minimal chance for cure: parent proxy of the child's health-related quality of life and the effect on parental physical and mental health during treatment. *Journal of Neuro-Oncology* **2016**, *129*, 373-381, doi:10.1007/s11060-016-2187-9.<br>Palmer, S.N.; Meeske, K.A.; Katz, E.R.; Burwinkle, T.M.; Varni, J.W. The PedsQL Brain Tumor Module: Initial reliability and validity. *Pediatr Blood Cancer* **2007**, *49*, 287-293, doi:10.1002/pbc.21026.<br>Sato, I.; Higuchi, A.; Yanagisawa, T.; Mukasa, A.; Ida, K.; Sawamura, Y.; Sugiyama, K.; Saito, N.; Kumabe, T.; Terasaki, M.; et al. Development of the Japanese version of the Pediatric Quality of Life

	Inventory™ Brain Tumor Module. *Health Qual Life Outcomes* **2010**, *8*, 38, doi:10.1186/1477-7525-8-38.<br>Sato, I.; Higuchi, A.; Yanagisawa, T.; Mukasa, A.; Ida, K.; Sawamura, Y.; Sugiyama, K.; Saito, N.; Kumabe, T.; Terasaki, M.; et al. Factors influencing self- and parent-reporting health-related quality of life in children with brain tumors. *Qual Life Res* **2013**, *22*, 185-201, doi:10.1007/s11136-012-0137-3.
PedsQL-Cancer	Banks, B.A.; Barrowman, N.J.; Klaassen, R. Health-related quality of life: Changes in children undergoing chemotherapy. *J Pediatr Hematol Oncol* **2008**, *30*, 292-297, doi:10.1097/MPH.0b013e3181647bda.<br>Felder-Puig, R.; Frey, E.; Proksch, K.; Varni, J.W.; Gadner, H.; Topf, R. Validation of the German version of the Pediatric Quality of Life Inventory (PedsQL) in childhood cancer patients off treatment and children with epilepsy. *Qual Life Res* **2004**, *13*, 223-234, doi:10.1023/B:QURE.0000015305.44181.e3.<br>Lau, J.T.; Yu, X.N.; Chu, Y.; Shing, M.M.; Wong, E.M.; Leung, T.F.; Li, C.K.; Fok, T.F.; Mak, W.W. Validation of the Chinese version of the Pediatric Quality of Life Inventory (PedsQL) Cancer Module. *J Pediatr Psychol* **2010**, *35*, 99-109, doi:10.1093/jpepsy/jsp035.<br>Santos, S.; Crespo, C.; Canavarro, M.C.; Fernandes, A.; Batalha, L.; de Campos, D.; Pinto, A. Psychometric study of the European Portuguese version of the PedsQL 3.0 Cancer Module. *Health Qual Life Outcomes* **2016**, *14*, 20, doi:10.1186/s12955-016-0421-y.<br>Scarpelli, A.C.; Paiva, S.M.; Pordeus, I.A.; Ramos-Jorge, M.L.; Varni, J.W.; Allison, P.J. Measurement properties of the Brazilian version of the Pediatric Quality of Life Inventory (PedsQL) cancer module scale. *Health Qual Life Outcomes* **2008**, *6*, 7, doi:10.1186/1477-7525-6-7.<br>Tsuji, N.; Kakee, N.; Ishida, Y.; Asami, K.; Tabuchi, K.; Nakadate, H.; Iwai, T.; Maeda, M.; Okamura, J.; Kazama, T.; et al. Validation of the Japanese version of the Pediatric Quality of Life Inventory (PedsQL) Cancer Module. *Health Qual Life Outcomes* **2011**, *9*, 22, doi:10.1186/1477-7525-9-22.<br>Varni, J.W.; Burwinkle, T.M.; Katz, E.R.; Meeske, K.; Dickinson, P. The PedsQL in pediatric cancer: Reliability and validity of the Pediatric Quality of Life Inventory Generic Core Scales, Multidimensional Fatigue Scale, and Cancer Module. *Cancer* **2002**, *94*, 2090-2106, doi:10.1002/cncr.10428.<br>Veldhuijzen Van Zanten, S.E.M.; El-Khouly, F.E.; Jansen, M.H.A.; Bakker, D.P.; Sanchez Aliaga, E.; Haasbeek, C.J.A.; Wolf, N.I.; Zwaan, C.M.; Vandertop, W.P.; Van Vuurden, D.G.; et al. A phase I/II study of gemcitabine during radiotherapy in children with newly diagnosed diffuse intrinsic pontine glioma. *Journal of Neuro-Oncology* **2017**, *135*, 307-315, doi:10.1007/s11060-017-2575-9.
PeNAT	Dupuis, L.L.; Taddio, A.; Kerr, E.N.; Kelly, A.; MacKeigan, L. Development and validation of the Pediatric Nausea Assessment Tool for use in children receiving antineoplastic agents. *Pharmacotherapy* **2006**, *26*, 1221-1231, doi:10.1592/phco.26.9.1221.<br>Dupuis, L.L.; Tamura, R.N.; Kelly, K.M.; Krischer, J.P.; Langevin, A.M.; Chen, L.; Kolb, E.A.; Ullrich, N.J.; Sahler, O.J.Z.; Hendershot, E.; et al. Risk factors for chemotherapy-induced nausea in pediatric

	patients receiving highly emetogenic chemotherapy. *Pediatr Blood Cancer* **2019**, *66*, e27584, doi:10.1002/pbc.27584.<br>Evans, A.; Malvar, J.; Garretson, C.; Pedroja Kolovos, E.; Baron Nelson, M. The use of aromatherapy to reduce chemotherapy-induced nausea in children with cancer: A randomized, double-blind, placebo-controlled trial. *J Pediatr Oncol Nurs* **2018**, *35*, 392-398, doi:10.1177/1043454218782133.<br>Flank, J.; Sparavalo, J.; Vol, H.; Hagen, L.; Stuhler, R.; Chong, D.; Courtney, S.; Doyle, J.J.; Gassas, A.; Schechter, T.; et al. The burden of chemotherapy-induced nausea and vomiting in children receiving hematopoietic stem cell transplantation conditioning: a prospective study. *Bone Marrow Transplantation* **2017**, *52*, 1294-1299, doi:10.1038/bmt.2017.112.<br>Loves, R.; Tomlinson, D.; Baggott, C.; Dix, D.; Gibson, P.; Hyslop, S.; Johnston, D.L.; Orsey, A.D.; Portwine, C.; Price, V.; et al. Taste changes in children with cancer and hematopoietic stem cell transplant recipients. *Supportive Care in Cancer* **2019**, *27*, 2247-2254, doi:10.1007/s00520-018-4509-2.
POQOLS	Barrera, M.; Gee, C.; Andrews, G.S.; Armstrong, C.A.; Saunders, F.E. Health-related quality of life of children and adolescents prior to hematopoietic progenitor cell transplantation: Diagnosis and age effects. *Pediatr Blood Cancer* **2006**, *47*, 320-326, doi:10.1002/pbc.20601.<br>Goodwin, D.A.J.; Boggs, S.R.; Graham-Pole, J. Development and validation of the Pediatric Oncology Quality of Life Scale. *Psychol Assess* **1994**, *6*, 321-328, doi:10.1037/1040-3590.6.4.321.<br>Kazak, A.E.; Penati, B.; Waibel, M.K.; Blackall, G.F. The Perception of Procedures Questionnaire: Psychometric Properties of a Brief Parent Report Measure of Procedural Distress. *Journal of Pediatric Psychology* **1996**, *21*, 195-207, doi:10.1093/jpepsy/21.2.195.<br>Mounir, G.M.; Abolfotouh, M.A. Assessment of health related quality of life among school children with cancer in Alexandria. *J Egypt Public Health Assoc* **2007**, *82*, 219-238.<br>Peeters, J.; Meitert, J.; Paulides, M.; Wiener, A.; Beck, J.D.; Calaminus, G.; Langer, T. Health-related quality of life (HRQL) in all-patients treated with chemotherapy only: A report from the late effects surveillance system in Germany. *Klin Padiatr* **2009**, *221*, 156-161, doi:10.1055/s-0029-1216366.
SSPedi	Dupuis, L.L.; Johnston, D.L.; Baggott, C.; Hyslop, S.; Tomlinson, D.; Gibson, P.; Orsey, A.; Dix, D.; Price, V.; Vanan, M.; et al. Validation of the Symptom Screening in Pediatrics Tool in children receiving cancer treatments. *J Natl Cancer Inst* **2018**, *110*, 661-668, doi:10.1093/jnci/djx250.<br>Hyslop, S.; Dupuis, L.L.; Baggott, C.; Dix, D.; Gibson, P.; Kuczynski, S.; Johnston, D.L.; Orsey, A.; Portwine, C.; Price, V.; et al. Validation of the proxy version of Symptom Screening in Pediatrics Tool in children receiving cancer treatments. *J Pain Symptom Manage* **2018**, *56*, 107-112, doi:10.1016/j.jpainsymman.2018.03.025.<br>Loves, R.; Tomlinson, D.; Baggott, C.; Dix, D.; Gibson, P.; Hyslop, S.; Johnston, D.L.; Orsey, A.D.; Portwine, C.; Price, V.; et al. Taste changes in children with cancer and hematopoietic stem cell

	transplant recipients. *Supportive Care in Cancer* **2019**, *27*, 2247-2254, doi:10.1007/s00520-018-4509-2.<br>O'Sullivan, C.; Dupuis, L.L.; Gibson, P.; Johnston, D.L.; Baggott, C.; Portwine, C.; Spiegler, B.; Kuczynski, S.; Tomlinson, D.; De Mol Van Otterloo, S.; et al. Refinement of the Symptom Screening in Pediatrics Tool (SSPedi). *Br J Cancer* **2014**, *111*, 1262-1268, doi:10.1038/bjc.2014.445.<br>Tomlinson, D.; Dupuis, L.L.; Gibson, P.; Johnston, D.L.; Portwine, C.; Baggott, C.; Zupanec, S.; Watson, J.; Spiegler, B.; Kuczynski, S.; et al. Initial development of the Symptom Screening in Pediatrics Tool (SSPedi). *Support Care Cancer* **2014**, *22*, 71-75, doi:10.1007/s00520-013-1945-x.
TRSC-C	Li, H.C.W.; Williams, P.D.; Williams, A.R.; Chung, J.O.K.; Chiu, S.Y.; Lopez, V. Confirmatory factor analysis of the Chinese version of the Pediatric Quality-of-Life Inventory Cancer Module. *Cancer Nurs* **2013**, *36*, E66-E72, doi:10.1097/NCC.0b013e318276e056.<br>Mansouri, A.; Motaghedi, R.; Rashidian, A.; Ashouri, A.; Kagrar, M.; Hajibabaei, M.; Gholami, K.; Ansari, S. Validity and reliability assessment of the Persian version of Therapy-Related Symptom Checklist. *Iran J Med Sci* **2017**, *42*, 292-300.<br>Williams, P.D.; Piamjariyakul, U.; Shanberg, R.; Williams, A.R. Monitoring and Alleviation of Symptom Occurrence and Severity Among Thai Children and Adolescents During Cancer Treatments. *Journal of Pediatric Oncology Nursing* **2015**, *32*, 417-428, doi:10.1177/1043454214563754.<br>Williams, P.D.; Schmideskamp, J.; Ridder, E.L.; Williams, A.R. Symptom monitoring and dependent care during cancer treatment in children: Pilot study. *Cancer Nurs* **2006**, *29*, 188-197, doi:10.1097/00002820-200605000-00004.<br>Williams, P.D.; Williams, A.R.; Kelly, K.P.; Dobos, C.; Gieseking, A.; Connor, R.; Ridder, L.; Potter, N.; Del Favero, D. A symptom checklist for children with cancer: The Therapy-Related Symptom Checklist-Children. *Cancer Nurs* **2012**, *35*, 89-98, doi:10.1097/NCC.0b013e31821a51f6.
